# SIRT1/PGC-1α/PPAR-γ Correlate With Hypoxia-Induced Chemoresistance in Non-Small Cell Lung Cancer

**DOI:** 10.3389/fonc.2021.682762

**Published:** 2021-07-26

**Authors:** Rui Xu, Xin Luo, Xuan Ye, Huan Li, Hongyue Liu, Qiong Du, Qing Zhai

**Affiliations:** ^1^ Department of Pharmacy, Fudan University Shanghai Cancer Center, Minhang Branch, Shanghai, China; ^2^ Department of Pharmacy, Fudan University Shanghai Cancer Center, Shanghai, China; ^3^ Department of Oncology, Shanghai Medical College, Fudan University, Shanghai, China

**Keywords:** non-small cell lung cancer, chemoresistance, SIRT1, PGC-1α, PPAR-γ

## Abstract

Resistance is the major cause of treatment failure and disease progression in non-small cell lung cancer (NSCLC). There is evidence that hypoxia is a key microenvironmental stress associated with resistance to cisplatin, epidermal growth factor receptor (EGFR) tyrosine kinase inhibitors (TKIs), and immunotherapy in solid NSCLCs. Numerous studies have contributed to delineating the mechanisms underlying drug resistance in NSCLC; nevertheless, the mechanisms involved in the resistance associated with hypoxia-induced molecular metabolic adaptations in the microenvironment of NSCLC remain unclear. Studies have highlighted the importance of posttranslational regulation of molecular mediators in the control of mitochondrial function in response to hypoxia-induced metabolic adaptations. Hypoxia can upregulate the expression of sirtuin 1 (SIRT1) in a hypoxia-inducible factor (HIF)-dependent manner. SIRT1 is a stress-dependent metabolic sensor that can deacetylate some key transcriptional factors in both metabolism dependent and independent metabolic pathways such as HIF-1α, peroxisome proliferator-activated receptor gamma (PPAR-γ), and PPAR-gamma coactivator 1-alpha (PGC-1α) to affect mitochondrial function and biogenesis, which has a role in hypoxia-induced chemoresistance in NSCLC. Moreover, SIRT1 and HIF-1α can regulate both innate and adaptive immune responses through metabolism-dependent and -independent ways. The objective of this review is to delineate a possible SIRT1/PGC-1α/PPAR-γ signaling-related molecular metabolic mechanism underlying hypoxia-induced chemotherapy resistance in the NSCLC microenvironment. Targeting hypoxia-related metabolic adaptation may be an attractive therapeutic strategy for overcoming chemoresistance in NSCLC.

## Introduction

Lung cancer is the most commonly diagnosed malignancy and the leading cause of cancer-related morbidity and mortality worldwide. Non-small cell lung cancer (NSCLC) accounts for approximately 80–85% of all histological subtypes of lung cancer (1, 2). The main treatment options include surgery, radiotherapy, and chemotherapy. Cisplatin, a potent DNA-damaging anticancer agent, remains a cornerstone for treating NSCLC, and its major pharmacological effect is to induce cancer cell apoptosis (3, 4). Targeted molecular therapy is increasingly recognized as a potent strategy in the treatment of NSCLC. Epidermal growth factor receptor (EGFR) is a tyrosine kinase receptor that meditates the proliferation, migration, survival, and apoptosis of epithelial cells. Primary mutations in the EGFR gene are the most common driver of lung cancer initiation and progression. EGFR mutations are detected in approximately 15% of all NSCLC patients and are associated with the development of this disease (5, 6). Targeted therapy with EGFR tyrosine kinase inhibitors (TKIs) has achieved superior efficacy in terms of progression-free survival and overall survival compared with conventional chemotherapy in NSCLC patients with EGFR mutation. Similarly, immunotherapy significantly prolongs survival in some advanced or locally advanced NSCLC and extensive SCLC in the past decade. Resistance to chemotherapies remain a major cause of treatment failure and disease progression in NSCLC patients (7).

## Different Mechanisms of Chemotherapy Resistance in NSCLC

**Figure 1:**
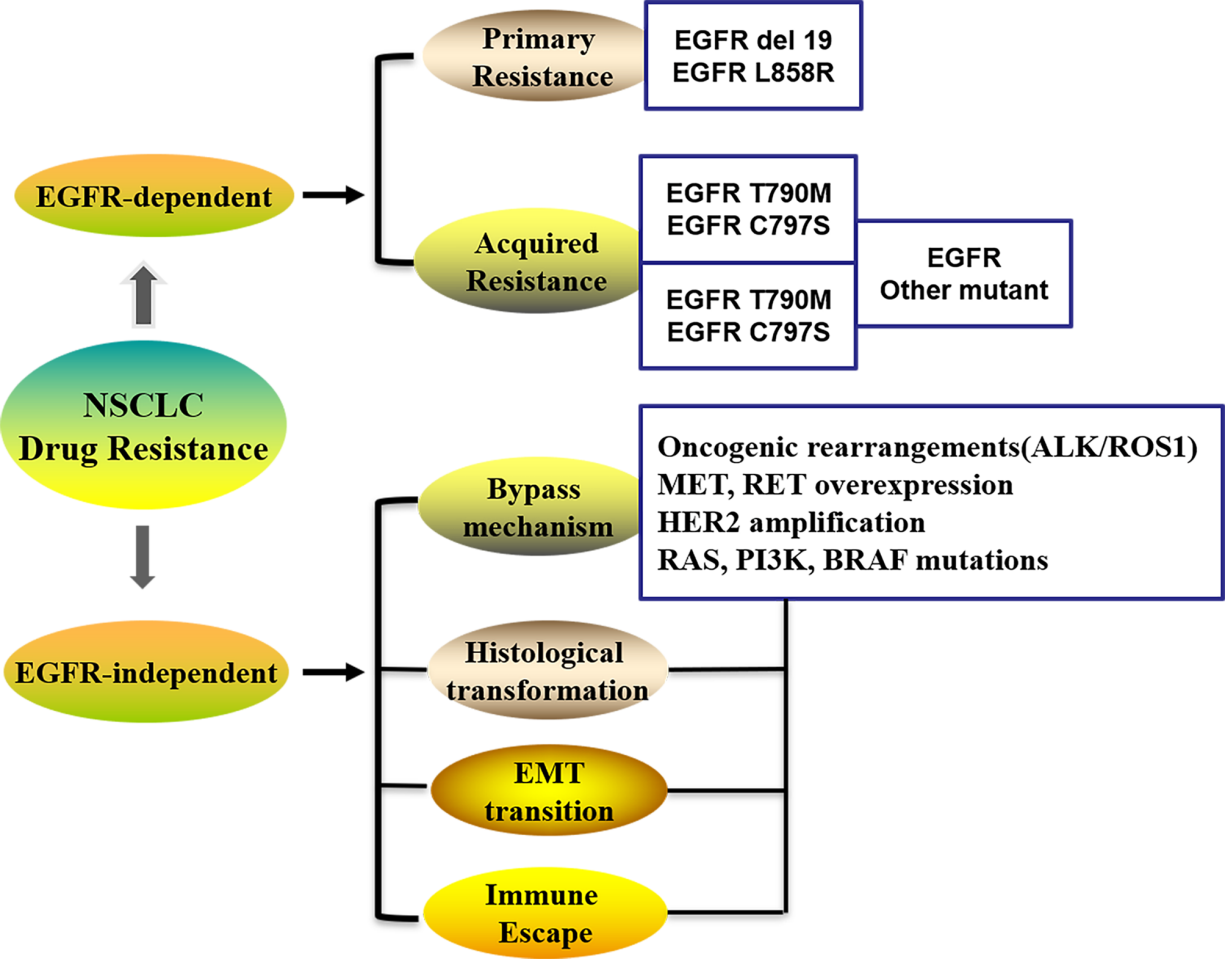
The patterns in the development of drug-resistance NSCLC. NSCLC, Non-small cell lung cancer; EGFR, Epidermal growth factor receptor; EMT, Epithelial–mesenchymal transition; ALK, Anaplastic lymphoma kinase; ROS1, receptor tyrosine kinase; Her-2, Human epidermal growth factor; PI3K, phosphatidylinositol‐3‐kinase; RAS, Rat sarcoma; MET, Proto-oncogene receptor tyrosine kinase; RET, Rearranged during transfection; BRAF, B-Raf proto-oncogene.

Several possibilities have been proposed to explain the mechanisms underlying drug resistance to chemotherapy in lung cancer (8–10), including alteration of drug transport; an improved detoxification ability of the tumor itself; increased DNA repair; regulation of resistance-related genes, the cell cycle, and cell death; effector T-cell infiltration in the TME; epigenetic modulation; and metabolic adaptations, et al. Drug efflux transporter from the ATP-binding cassette (ABC) family and the P-glycoprotein (P-gp) is associated with the expression of multidrug resistance, which contribute to chemotherapy failure by extruding drugs from tumor cells to the extracellular compartments (11). The patterns of resistance in 1st and 2nd generation EGFR-TKIs are largely overlapping and primarily involved activation of the MAPK-PI3K pathway, cell cycle gene alterations, rearrangements of RET or ALK kinases, and many other genomic alterations, which lead to primary and acquired resistance against EGFR-TKIs in lung cancer (**Figure 1**) (12). Crucially, secondary acquired mutations in EGFR account for approximately 50% of all cases of EGFR-TKI-related resistance in NSCLC patients (13, 14). The complexity of the mechanisms associated with acquired EGFR-TKI resistance in NSCLC has been widely demonstrated (15), and can be grouped into kinase domain mutations and overexpression of target oncogenes within tumor cells (9). The EGFR T790M mutation is the primary cause of drug resistance in 50–60% of instances, while activating genomic alterations in other kinases, such as ALK, ROS1, MET, RET, NTRK, and BRAF, have been validated as targets in NSCLC therapy (**Figure 1**) (16). It is evident that secondary (T790M) or tertiary (C797S) mutations are the main factors responsible for the development of acquired resistance (17).

Furthermore, resistance to immunotherapy in lung cancer manifests as a lack of initial response or clinical benefit to therapy (primary resistance) or tumor progression after the initial period of response (acquired resistance). The primary resistance prevents the infiltration or function of immune cells in the TME, while Loss of T cell function may be a potential mechanism for acquired resistance to immunodetection point inhibitors (18, 19). In addition, metabolic properties of non-small-cell lung cancers can reprogram stromal cells to induce resistance to EGFR inhibitors (11, 20). Cellular metabolism leads to a tumor microenvironment (TME) that is commonly acidic, hypoxic and/or depleted of critical nutrients required by immune cells, which can alter the antitumor immune response and even promote resistance to immunotherapy.

Hypoxia-related stress is a prominent microenvironmental feature in solid tumors and is associated with angiogenesis and acquired resistance to cancer chemotherapy. The hypoxic environment in solid tumors results from, among other factors, the rapid proliferation of tumor cells, leading to the depletion of available oxygen (21, 22). Reduced oxygen levels in tumor tissues result in the stabilization and accumulation of HIF-1α, which plays a key role in the adaptive response of cancer cells to hypoxia by modulating various cellular functions (23). Hypoxia is widely associated with promoting chemoresistance in tumor cells and maybe a potential target for NSCLC therapy. EGFR overexpression and tumor hypoxia have been shown to correlate with worse outcome in several types of cancer (24). However, little is known about the mechanisms of resistance associated with hypoxia-induced epigenetic changes and molecular metabolic flexibility in the microenvironment of drug-resistant NSCLC (25). A greater understanding of the underlying molecular mechanisms of chemoresistance is still needed to allow the elaboration of strategies to overcome drug resistance (26). Herein, we summarize the literature relating to the development of hypoxia-induced chemotherapy resistance in NSCLC, focusing on the putative epigenetic and metabolic microenvironmental mechanisms.

## Hypoxia-Induced Chemotherapy Resistance in NSCLC

**Figure 2:**
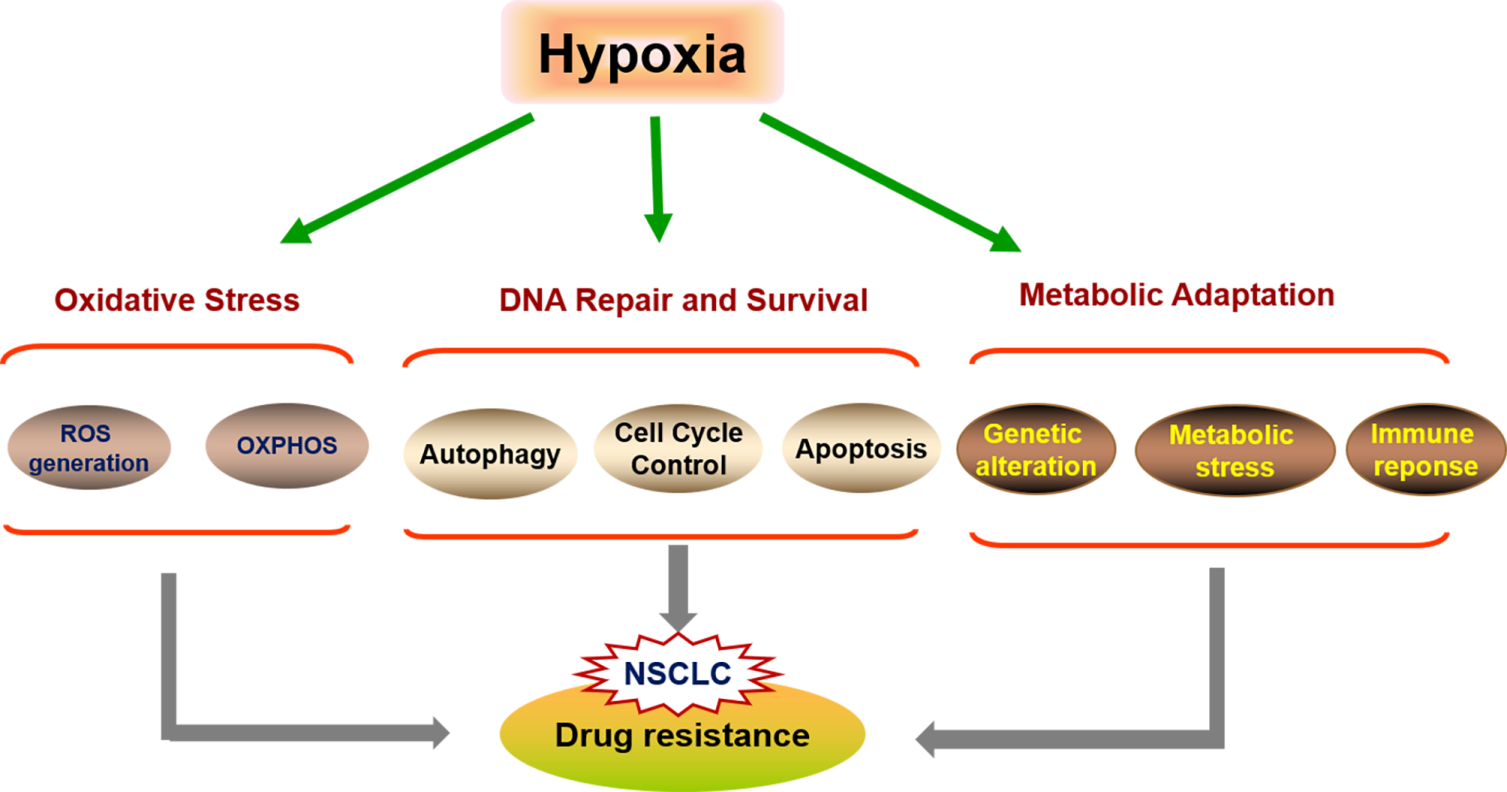
The underlying mechanisms of hypoxia-induced drug-resistant NSCLC. Tumors exposed to hypoxia is an important mediator of resistance to cisplatin and EGFR-TKIs in NSCLC that induce apoptosis-resistant pathways by mediating autophagy and reducing susceptibility to apoptosis, and then metabolic adaptations in tumor cells to hypoxia leads to chemoresistance, which switches on genetic pathways and metabolic changes.

Hypoxia is an important factor in treatment resistance and poor survival. Numerous studies have shown that hypoxia in the tumor microenvironment is an important mediator of resistance to chemotherapy through activating signaling pathways and inducing metabolic changes (**Figure 2**). Hypoxia can affect drug delivery, DNA repair, the regulation of resistance-related genes, the cell cycle, and cell death-associated pathways, thereby promoting chemoresistance and tumor malignancy (27–30). The most likely explanation for hypoxia-induced cisplatin resistance is a reduced cellular susceptibility to apoptosis (31, 32). Recent studies have revealed that hypoxia can modulate autophagy, thereby increasing cell survival and chemoresistance (33, 34), including in lung cancer (35). Studies have suggested that hypoxia can augment cisplatin-induced autophagy, and hypoxia-induced autophagy contributes toward chemoresistance in NSCLC cells (31, 35). EGFR-TKIs are reported to increase hypoxia-induced autophagy and promote cell death (36, 37). Crucially, hypoxia induces Hypoxia-inducible factors (HIFs) stabilization and downstream signaling that play key roles in cellular responses to hypoxia. The effect of hypoxia on cisplatin resistance is mediated, at least in part, through HIF-1α and P53. Exposure to hypoxia was shown to induce HIF-1α and P53 expression and promote reactive oxygen species (ROS) generation and glycolysis in NSCLC A549 cells (38–40). High glycolytic tumor metabolism can suppress immune function and mediate tumor immune escape, which is associated with resistance to chemotherapy (41). Recent experiments using tumor cell lines also support that hypoxia-induced HIF-1α can increase PD-L1 expression on tumor cells, *via* direct interaction with a hypoxia-response element in the PD-L1 proximal promoter to activate transcription (42). Hypoxia may also be an important mediator of resistance to EGFR-TKIs *via* the upregulation of FGFR1 and the MAPK pathway in the NSCLC cell line H1975 (43).

A major consequence of EGFR signaling in hypoxic tumors appears to be an enhanced induction of vascular endothelial growth factor (VEGF); and the molecular mechanisms underlying the link between EGFR signaling and VEGF expression include both HIF-1α–dependent and independent mechanisms. VEGF, a HIF-1α target gene, is modulated by activation of several receptor tyrosine kinases, and EGFR inhibition has been found to decrease VEGF expression in many tumor types (44). HIF-1α transcriptionally regulates VEGF expression and binds directly to the hypoxia-response elements in the promoters of the VEGF-regulated genes. EGFR has been implicated as a hypoxia-independent driver of HIFs expression as inhibition of EGFR activation has been reported to reduce HIF-1α protein translation in some cells. Recently, a research has indicated that EGFR-mutant NSCLC cells display a HIF-1α and VEGF-dependent phenotype and that in EGFR-mutant NSCLC cells, EGFR, but not hypoxia, regulates HIF-1a transcription and protein stability, whereas in EGFR WT NSCLC, hypoxia is the primary regulator of HIF-1α. Moreover, cells with acquired EGFR inhibitor resistance retained elevated expression of HIF-1α and VEGF, and the pathways were uncoupled from EGFR-regulated (45). Meanwhile, VEGF expression within the TME is heterogenous and mainly hypoxia-driven, which also exerts immunosuppressive effects (46).

In addition, one study has reported that microRNAs, metabolic pathways, and pseudohypoxia initiate drug tolerance to EGFR inhibitors in lung adenocarcinoma (47). Lu et al. demonstrated that prolonged, long-term, moderate hypoxia can promote resistance to the third-generation EGFR-TKI osimertinib (AZD9291) in the NSCLC cell line H1975 that had developed resistance to first- and second-generation EGFR-TKIs *via* the T790M EGFR mutation (43, 48). Hypoxia exposure induces gefitinib resistance in both EGFR-wild type and EGFR-mutated NSCLC through epigenetic changes and regulation of epithelial–mesenchymal transition (EMT) (49–51). Similarly, it was shown that hypoxia can significantly reduce chemosensitivity and induce multidrug resistance in NSCLC cells *via* the enhancement of epidermal growth factor-like domain 7 (EGFL7) expression (52). Hypoxia induces chemoresistance through adaptive metabolic changes involving pleiotropic mechanisms that are associated with significant intra-tumor metabolic heterogeneity (53). Several studies have proposed that tumor histology and local microenvironment influence metabolic adaptation, while certain epigenetic modification and tumor plasticity confer differential sensitivity to metabolic interventions. Therefore, precision targeted therapy efficacy could be improved by stratify certain types of cancer into molecular and metabolic subtypes (54). There is some evidence to support preclinical and pilot clinical investigation of combining EGFR-TKI with hypoxia-targeted therapies in EGFR-mutated NSCLC. Interestingly, determinants of tumor hypoxia status, such as *in vivo* oximetry, gene expression signatures, PET imaging of hypoxia, and circulating biomarkers, can predict therapeutic resistance to a wide array of oncological treatments (55). Furthermore, *in vitro* 3D models mimicking NSCLC hypoxia have shown promise in helping to elucidate the pathogenesis of lung cancer and identifying potential therapeutic targets (56).

In brief, hypoxia-targeted therapies have the potential to help overcome chemoresistance in NSCLC (**Figure 2**). Next, we aim to delineate a possible molecular mechanism underlying hypoxia-induced chemotherapy resistance involving hypoxia-associated mitochondrial and nuclear energy metabolism-related genes that could be clinically relevant and therapeutically exploitable.

## Sirtuin 1 (SIRT1) Correlates With the Occurrence and Development of NSCLC

The loss of homeostasis in the acetylation of histone and nonhistone proteins is closely related to tumor occurrence and development, and represents a potential target for cancer therapy (57, 58). SIRT1, a nicotinamide adenine dinucleotide-dependent (NAD) deacetylase, is a recently identified epigenetic regulator that can deacetylate histone and nonhistone proteins, including transcription factors. Accumulating evidence has supported that SIRT1 is involved in tumorigenesis and cancer development (59). However, the role of SIRT1 in cancer progression and therapeutic responses remains controversial because SIRT1 has both tumor-promoting (60) and tumor-suppressing functions (61), depending on whether oncogenes or tumor-suppressor genes are targeted (62). The overexpression of SIRT1 promotes the progression of various solid tumors such as lung cancer, breast cancer, ovarian cancer, gastric cancer, colon cancer, and esophageal squamous cell carcinoma, and is an indicator of poor prognosis (63–65). Relevant findings have suggested that SIRT1 expression is higher in NSCLC tissue than in surrounding normal tissues. SIRT1 overexpression plays a promotive role in tumorigenesis and is closely associated with tumor invasion and lymph node metastasis in NSCLC (66, 67). Meanwhile, the prognosis, overall survival, and disease-free survival of NSCLC patients with high SIRT1 expression were significantly worse than those of NSCLC patients with low SIRT1 expression (68). A study of the correlation between SIRT1 and the clinical characteristics of NSCLC patients revealed that SIRT1 tends to be highly expressed in poorly differenced cancers, indicating that it plays a tumorigenic role in NSCLC (69, 70).

The targets of SIRT1 and the related signaling pathways in tumors have also been investigated in-depth. SIRT1 has been reported to regulate apoptotic, inflammatory, and oxidative stress-related processes in ischemia/hypoxia through the deacetylation of its downstream targets, including P53, nuclear factor-kappa B (NF-κB), PGC-1α, forkhead box Os (FOXOs), and PPARs (71, 72). The overexpression of SIRT1 can inhibit P53-regulated cell cycle arrest under conditions of DNA damage and oxidative stress (73). Similarly, the activation of the SIRT1/AMPK signaling pathway can inhibit the proliferation and migration of the human NSCLC cells A549 and H1299 (74). When compared with the control group, the abnormal expression of SIRT1 and AMPK were shown to differ significantly between the NSCLC group and controls, and this differing expression was related to NSCLC occurrence and development (75). FOXOs are involved in the regulation of apoptosis, the cell cycle, and DNA damage repair and their deacetylation by SIRT1 inhibits their transcriptional and biological activity (76, 77).

Certainly, Studies have supported that SIRT1 plays a promotive role in the acquisition of chemoresistance in tumors (78, 79). Analysis of the relevant literature indicates that SIRT1 overexpression can enhance tumor resistance to therapy, possibly by reducing the penetration of drugs into cells, promoting the acquisition of drug resistance through genetic mutations, or changing the tumor microenvironment (80–84). SIRT1 is a key anti-apoptotic factor in tumor cells, and SIRT1 activity was found to be activated by chemotherapeutic agents in certain tumor cell lines. SIRT1 has been reported to regulate neovascularization by reducing the transcriptional activity of P53 by deacetylation of its lysine residues (85, 86). A study investigating the effects of SIRT1 activators and inhibitors on CD44+/CD133+-enriched NSCLC cells reported that SIRT1 can deacetylate the tumor suppressor protein P53, thereby decreasing its activity (87). Meanwhile, the modulation of SIRT1 expression by resveratrol promoted the collateral sensitivity of drug-resistant ABCB5- and mutation-activated EGFR overexpressing cells (88). Both genetic and chemical inhibition of SIRT1 can reverse chemoresistance in lung cancer cells by enhancing DNA damage and activating apoptosis, concomitant with XRCC1 degradation (82). Gong et al. recently also confirmed that NSCLC patients with high SIRT1 expression have a significantly higher rate of resistance to chemotherapy than those with low SIRT1 expression (89). Additionally, high SIRT1 expression can induce resistance to cisplatin in A549 cells *via* modulating Noxa expression (90).

Studies on the antitumor effects of EGFR-TKIs or EGFR-TKIs combined with HDAC inhibitors on NSCLC have also demonstrated that HDAC inhibitors decrease the survival rates of A549, hcc827, and hcc827ir cells, and enhance the sensitivity of EGFR-TKI-resistant cell lines to EGFR-TKIs through synergistic effects (91, 92). Interestingly, SIRT1 can promote the acquisition of stem cell characteristics in tumor cells, and these cells can become resistant to chemotherapy, radiation, and target drugs, and plays a key role in malignant progression of tumors, tumor metastasis, and cancer recurrence (93). When compared with their parental cells, cancer stem cells have an increased ability to develop resistance to EGFR-TKIs in NSCLC; EGFR-TKI-resistant sublines with stem cell-like properties are also resistant to conventional chemotherapeutic drugs, but equally sensitive to histone deacetylase and proteasome inhibitors (94, 95). In EGFR-TKI-resistant xenograft models, the combined administration of a SIRT1 inhibitor, tenovin-6 (Tv6), with gefitinib promoted tumor regression. Additionally, Tv6/gefitinib coadministration leads to a decrease in the dose of gefitinib necessary to induce tumor responses in preclinical models (96). This indicates that SIRT1 plays a vital role in the regulation of the NSCLC microenvironment (97), and targeting SIRT1 might represent a potential therapeutic strategy for the treatment of drug-resistant NSCLC.

## The Association of SIRT1 With Hypoxia-Induced Chemoresistance in NSCLC

The hypoxic conditions prevailing in solid tumors can contribute to treatment failure and bad prognosis. Hypoxia is an important factor in treatment resistance and poor survival in NSCLC. Studies using different tumor cell lines, including NSCLC lines, have shown that hypoxia induces the expression of EGFR and its ligands. In turn, EGFR acts as a key survival factor under hypoxic conditions by enhancing cellular responses to hypoxia through increased expression of HIF-1α (98). There are several explanations for hypoxia-induced resistance in NSCLC. On the one hand, hypoxia can induce apoptosis resistance-related pathways by mediating autophagy and reducing susceptibility to apoptosis. On the other hand, metabolic adaptations to hypoxia in tumor cells, such as altered genetic pathways and metabolic stress, can result in chemoresistance (53). HIF-1α is central to the regulation of oxygen homeostasis and redox-sensitive metabolism, facilitating effective adaptation to hypoxia. SIRT1, a direct downstream target of HIF-1α, is a critical regulator of endothelial cell behavior and has been linked to tumor angiogenesis under hypoxic conditions (99). HIF-1α can be regulated by several upstream factors, including SIRT1, and metabolites, to regulate immune responses. The expression of SIRT1 is closely associated with the proliferation, survival, and resistance of many types of malignant tumors. Studies have shown that SIRT1 plays important roles in DNA damage responses, autophagy, and the maintenance of genome stability (100).

However, SIRT1 not only affects intracellular homeostasis, but also participates in the remodeling of the extracellular microenvironment (101). SIRT1 and HIF-1α, as metabolic sensors of redox and oxygen, can modulate both innate and adaptive immune responses through metabolism-dependent and -independent ways (102). Extensive evidence supports that, under hypoxic conditions, SIRT1 overexpression stabilizes HIF-1α *via* direct binding and deacetylation, which may be a prerequisite for a consequent enhancement of cell invasion (103, 104). It was demonstrated that in some cases, the effects of SIRT1 associated metabolism on innate immune cells is mediated by HIF-1α. SIRT1 and HIF-1α dependent metabolism is closely linked to adaptive immune responses induced under hypoxic conditions (105). When the NAD+/NADH balance is perturbed by ROS and oxidative stress under hypoxia, SIRT1 can regulate immune responses directly through deacetylation of some key transcriptional factors, such as P53, NF-kB, PGC-1α and PPAR-γ or indirectly regulate immune cell metabolism and response in both metabolism dependent and independent metabolic pathways (105).

SIRT1 has been reported to be involved in the regulation of various important biological processes, which can activate several transcription factors, such as PGC-1α and HIF-1α, resulting in ameliorated mitochondria biogenesis (106). Similar results on NSCLC cell lines grown under hypoxic conditions have revealed a novel mechanism of RBM38-mediated regulation of the HIF1α/miR-34a/SIRT1/P53 axis (107). Additionally, SIRT1 may be involved in the regulation of multiple aspects of tumor resistance by modulating the adaptive response of tumor cells (47–49). Hypoxia-mediated inactivation of the SIRT1/AMPK pathway led to cisplatin and doxorubicin resistance, indicating that this may be a strategy to overcome hypoxia-induced chemoresistance in NSCLC (78). One study showed that SIRT1 promotes the pro-apoptotic activity of the acetylated transcription factor P53 in A549 cells, while apoptosis is suppressed in cisplatin-resistant cells. The authors proposed that cytoplasm-localized SIRT1 downregulation may represent a novel therapeutic target to inhibit cisplatin resistance in cisplatin-resistant NSCLC cells (108). A review of the topic of histone deacetylase inhibition in NSCLC indicated that a SIRT1-mediated survival advantage may represent another mechanism through which NSCLC cells develop resistance to EGFR-TKIs (109), possibly through mediated by regulating the oxidative phosphorylation of mitochondria in lung adenocarcinoma cells, and then selectively eliminating TKI-resistant cancer stem cells.

Here, we discuss multiple mechanisms underlying hypoxia-induced chemotherapy resistance involving SIRT1-mediated deacetylation modifications of downstream transcription factors and the modulation of metabolic adaptation in the NSCLC microenvironment. We also consider how targeting SIRT1 has become a new therapeutic strategy linked with the hypoxic microenvironment of drug-resistant NSCLC.

## PGC-1α/PPAR-γ Signaling in Hypoxia-Induced Chemoresistance in NSCLC

**Figure 3:**
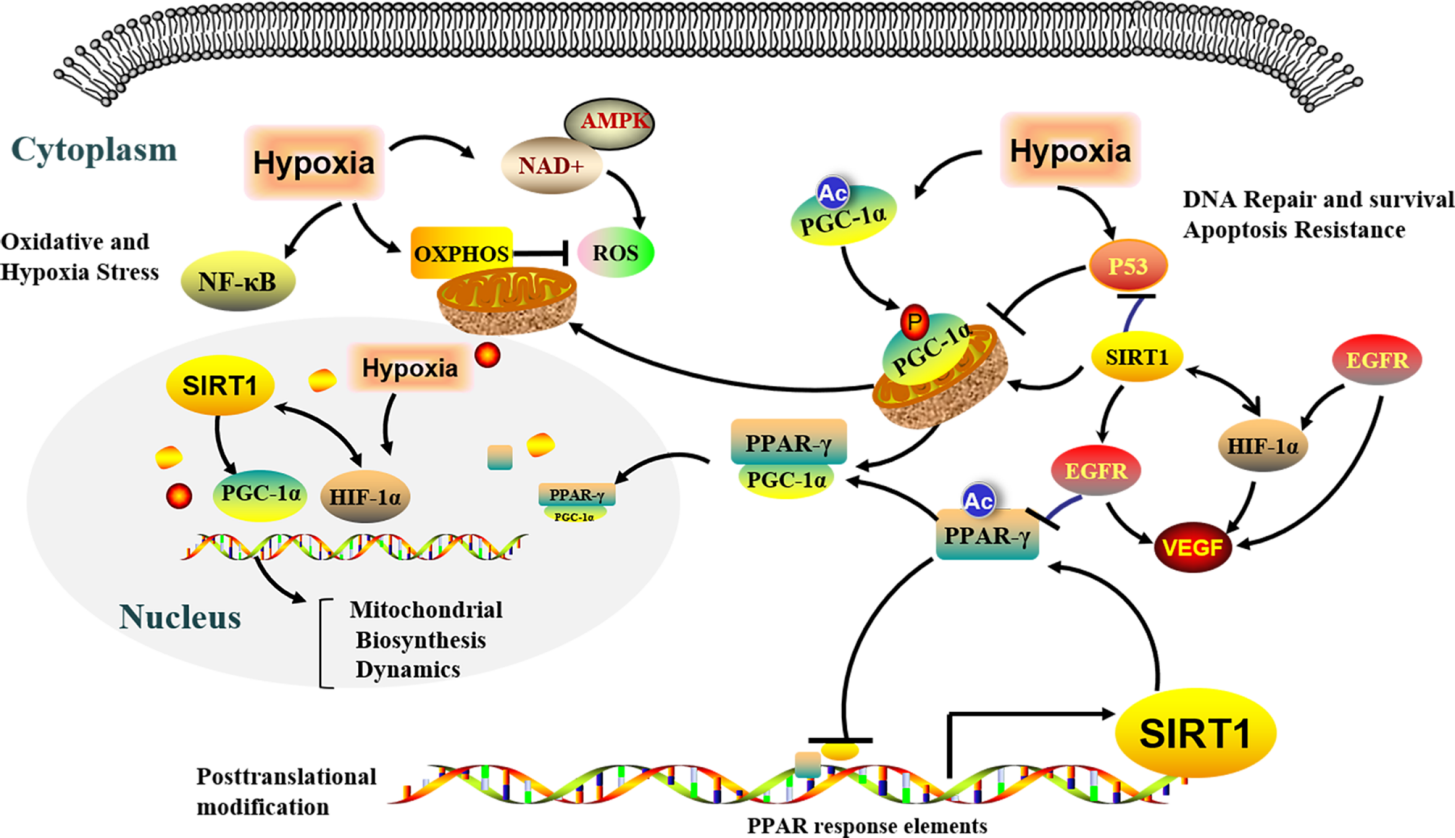
Regulation of metabolic microenvironment *via* SIRT1/PGC-1α/PPAR-γ signaling pathways under hypoxia conditions. Several potentially effective molecular mechanisms of regulation of metabolic microenvironment have been shown in non-small cell lung cancer under hypoxia conditions, by which SIRT1 mediated deacetylation modified of downstream transcription factors and modulated metabolic adaptation. SIRT1, Silent information regulator 1; PGC-1α, Peroxisome proliferator—activated receptor-γ coactivator-1α; PPAR-γ, Peroxisome proliferator activated receptor-γ; EGFR, Epidermal growth factor receptor; VEGF, Vascular endothelial growth factor; HIF-1α, Hypoxia inducible factor-1alpha; OXPHOS, Oxidative phosphorylation; ROS, Reactive oxygen species; NAD+, Nicotinamide adenine dinucleotide; AMPK, Adenosine monophosphate activated protein kinase.

Responses to hypoxic stress involve cellular adaptations in protein synthesis, energy metabolism, mitochondrial respiration, and nutrient acquisition (110, 111). Mitochondria are the main oxygen consumers at the crossroads of apoptotic pathways induced by anticancer agents, and excess mitochondrial activity leads to local hypoxia in the tumor microenvironment (**Figure 3**) (112, 113). Hypoxia may promote mitophagy, thereby mediating resistance to cisplatin (114), while mitophagy during hypoxia may limit ROS production (115, 116). Hypoxic cells in NSCLC are also more resistant to chemotherapeutics and radiation. Aggressive cancers become resistant to most chemotherapeutic drugs owing to the presence of clusters of drug-resistant cancer stem cells that exhibit an altered metabolic profile (117). A number of studies highlighted the importance of mitochondria-dependent metabolic reprogramming in the appearance of tumor evasion mechanisms and develop drug resistance (118). A novel mechanism of intercellular communication based on a horizontal transfer of mitochondria between non-tumor and malignant cells was described, which showed the phenomenon of direct mitochondria sharing could also contribute to resistance to existing drug combinations and possibly further promote tumor growth (118–120).

PGC-1α is a nuclear transcriptional coactivator of nuclear receptors and other transcription factors, and is also a master regulator of mitochondrial biogenesis necessary for efficient mitochondrial transfer. PGC-1α can promote mitochondrial biosynthesis, which includes the regulation of mitochondrial protein synthesis and the replication of mitochondrial genes (121, 122). PGC-1α levels have been reported to correlate with survival in patients with stage III NSCLC (123). Recent studies have suggested that the regulatory relationship between P53 and PGC-1α represents an important drug resistance mechanism in NSCLC. An analysis of gene expression in 28 human lung adenocarcinoma cell lines with different P53 mutational statuses reported that the suppression of PGC-1α inhibits the growth of lung adenocarcinoma cells with wild-type P53 (124). Meanwhile, low P53 expression and high PGC-1α expression correlated with poor sensitivity to cisplatin and apoptosis in NSCLC patients. Furthermore, P53 affects mitochondrial biosynthesis by regulating the stability of PGC-1α, thereby reducing NSCLC chemoresistance (125).

PPAR-γ is thought to function as a ligand-activated nuclear transcription factor. The activation of PPAR-γ and the subsequent regulation of the transcription of various genes can be directly mediated by the binding of ligands and molecules (126), which leads to heterodimerization with SIRT1 and the subsequent binding of the heterodimers to peroxisome proliferator response elements (PPREs) (127, 128). Accumulated evidence has demonstrated that PPAR-γ can also be activated through other mechanisms, including SIRT1-mediated deacetylation. Preclinical studies indicated that PPAR-γ can function as a tumor suppressor and play an important role in cell proliferation, cell differentiation, and apoptosis through a variety of mechanisms (129–133). The activation of PPAR-γ was also reported to inhibit lung tumor progression (134–137). Moreover, PPAR-γ is highly expressed in NSCLC, while PPAR-γ expression is highly correlated with tumor histological type, pathological differentiation status, and clinical stage (138–140). The expression of several tumor-associated genes was reported to be triggered by the inhibition of PPAR-γ activity through EGFR-mediated PPAR-γ phosphorylation and degradation, which, in turn, promoted nuclear EGFR/NF-κB signaling activation (141).

In NSCLC, PPAR-γ ligands (thiazolidinediones, such as rosiglitazone and pioglitazone) can promote tumor differentiation and inhibit tumor growth, metastasis, and angiogenesis (138, 142). Investigation of the interplay between troglitazone and chemotherapeutic drugs (cisplatin or paclitaxel) in xenotransplantation models indicated that chemotherapeutic drugs can induce PPAR-γ; moreover, the authors identified a sequence-specific synergy between PPAR-γ ligands and chemotherapeutic agents in the treatment of lung cancer (143). PPAR-γ activation may also protect cells against cisplatin toxicity by reducing metallothionein expression, proteins that promote resistance to cisplatin therapy. Additionally, PPAR-γ agonists synergistically increase the antitumor activity of gefitinib. For instance, Ni et al. showed that PPAR-γ activation can inhibit the proliferation of EGFR-TKI-resistant lung adenocarcinoma cells and lead to a better survival rate (135). Increasing evidence has indicated that PPAR-γ agonists may serve as master modulators for overcoming classic obstacles associated with targeted NSCLC therapies, including resistance to therapy and tumor genetic heterogeneity (144–146). These examples strongly suggest that PPAR-γ has potential as a molecular target for NSCLC treatment (129, 147–149).

Hypoxia-induced chemoresistance through a variety of mechanisms is associated with the metabolic signature of hypoxic cancer cells (**Figure 2**). The levels of PPAR-γ are influenced by changes in oxygen tension, which plays a critical role in the regulation of metabolism in cancers. Under hypoxic conditions, PPAR-γ expression can be induced *via* HIF-1α-dependent mechanisms (150). Hypoxia-induced repression of PPAR-γ can promote chemoresistance in NSCLC through the downregulation of uncoupling protein 2 (151). Cruz-Bermúdez et al. also reported a novel metabolic reprogramming-based cisplatin-resistance mechanism in NSCLC cells involving increased PGC-1α expression, oxidative metabolism, and mitochondrial biogenesis (152). Several studies have confirmed that some drug-resistant tumor cells are particularly strongly dependent on PGC-1α-mediated metabolic activity, and the inhibition of PGC-1α expression sensitizes some tumor cells to treatment, including lung cancer cells (153). The expression of a subset of known PGC-1α-regulated genes appears to be altered indirectly by changing the metabolic state of cells exposed to hypoxia and nutrient deprivation (154). Interestingly, PGC-1α is a regulator of PPAR-γ activity, and the dysregulated expression of PGC-1α may affect PPAR-γ function. In the late stage of tumor progression, PGC-1α is also considered to be an auxiliary activator of PPAR-γ and is closely involved in the resistance of tumors to chemotherapy (155).

Furthermore, one of the physiological consequences of hypoxia is reduced mitochondrial oxidative phosphorylation (OXPHOS). The activation of PGC-1α stimulates mitochondrial biogenesis and OXPHOS promoting cell mobility and metastases. High levels of PGC-1α are associated with increased OXPHOS-dependent metabolism in cells, while PGC-1α can also mediate accelerated mitochondrial OXPHOS and glycolysis in the heterogeneous tumor microenvironment (156, 157). Additionally, a study investigating the development of NSCLC resistance to EGFR-TKIs found that gefitinib-resistant NSCLC cells acquire metabolic flexibility characterized as a ligand-independent translocation of EGFR to mitochondria, which might contribute to the upregulation of mitochondrial function and capacity for OXPHOS (158). These observations indicate that the dependence on mitochondrial OXPHOS is a recurrent mechanism of cancer resistance to cisplatin treatment, while PGC-1α-mediated enhancement of mitochondrial biogenesis and OXPHOS is crucial for the development of chemoresistance in NSCLC under hypoxic conditions. Therefore, that PGC-1α/PPAR-γ have important roles in metabolic regulation, apoptosis, and drug resistance suggests that nuclear genes may be involved in hypoxia-induced resistance to chemotherapy through regulating mitochondrial biogenesis in the NSCLC microenvironment. Mitochondrial-dependent metabolic also underlines the significance of tumor microenvironment and cellular plasticity in cancer progression and drug resistance.

## SIRT1/PGC-1α/PPAR-γ in Hypoxia-Induced Chemoresistance in NSCLC

The resistance of NSCLC to therapy is mediated by several factors, among which hypoxia may be a key player. The acetylation of transcription factors, independently of histone modifications, has a central role in tumorigenesis and subsequent drug resistance. SIRT1, in addition to serving as an intermediary in cellular metabolism in gene silencing and aging, also functions as a pivotal regulator of various intracellular biological processes, including energy metabolism, DNA damage responses, the maintenance of genome stability, and tumorigenesis (159). SIRT1 may be associated with the susceptibility of the elderly to hypoxic injury, which leads to cell death *via* energy depletion and increased oxidative stress (160). Metabolic stress and biosynthetic stress are key factors affecting the NAD+ pool and NAD+-dependent SIRT1 activity (161). In the context of hypoxia-induced chemoresistance, SIRT1-dependent deacetylation may be primarily related to its ability to target and modulate the activity of signal transduction pathways and transcription factors such as P53, PPARs, PGC-1α, AMPK, FOXO proteins, and NF-κB (99, 162, 163). A mechanistic study in senescent WI-38 cells and animal tissues confirmed that both PPAR-γ and SIRT1 can bind to PPREs, which can be interpreted directly with SIRT1 to exert transcriptional activation in a ligand-dependent manner, partly by deacetylation (164). The inhibition of the SIRT1/PGC-1α/NRF2 pathway *via* the suppression of PPAR-γ transcriptional activity was reported to increase the susceptibility of wild-type P53-harboring cancer cells to oxidative stress and therapeutic agents (165). Among the targeting agents, studies have shown that the PPAR-γ agonist rosiglitazone facilitated the antiproliferative effects of gefitinib by upregulating PTEN expression in NSCLC A549 cells (166), while combination differentiation-based therapy involving PPAR-γ ligands and HDAC inhibitors enhanced the growth inhibition of lung adenocarcinoma cells (167).

The mitochondrion is an emerging therapeutic target in tumor cells (168), especially under hypoxic conditions. Cellular hypoxia can significantly decrease the expression of mitochondrial genes (169, 170) and induce metabolic adaptations in cancer cells (171). Tumor oxygenation responses to EGFR-TKI therapy appear to be mediated *via* effects on the hypoxia-regulated transcription factor HIF-1α and downstream expression of VEGF (37). Relevant research suggested that drug-resistent NSCLC of hypoxia-driven is associated with expression of HIF-1α and VEGF; and in tumors driven primarily by the EGFR pathway, targeting HIF or key HIF-regulated genes may further enhance the effect of EGFR inhibition alone and delay drug resistance (45). Similarly, high HIF-1α expression is associated with acquired resistance to EGFR-TKIs in NSCLC (172). These findings support hypoxia targeting in EGFR mutant tumors in the EGFR inhibitor–naive and refractory settings, which is the potential value of clinical testing and use of VEGF inhibitors in combination with EGFR-TKIs, but not just lung cancer. In response to hypoxic conditions, the posttranslational regulation of molecular mediators such as SIRT1, HIF-1α, PGC-1α, and AMPK may play a critical role in the control of the glycolytic-mitochondrial energy axis (**Figure 3**) (171, 173). SIRT1 and PGC-1α are supposed to exist in the mitochondria of tumorigenesis, where they function as critical inducible factors for intercellular energy metabolism and gene regulation (154, 174). SIRT1 is known to promote mitochondrial biogenesis. Considering to SIRT1, known as a modulator of PGC-1α activity, and is associated with metabolism and transcriptional responses through its NAD-dependent activity (175–177). Indeed, PGC-1α is a substrate of SIRT1, and regulates the activity of antioxidant enzymes and mitochondrial biosynthesis (178). Resveratrol, a well-known potent activator of SIRT1, has been shown to increase mitochondrial numbers by activating the SIRT1/PGC-1α pathway and inhibit VEGF induction through HIF-1α in ovarian tumor cells under hypoxic conditions (154).

Interestingly, numerous studies have demonstrated that an increase in SIRT1-mediated mitochondrial biogenesis plays prominent roles through both PGC-1α-dependent and PGC-1α-independent pathways. The SIRT1-mediated deacetylation of specific lysine residues activates PGC-1α, which then promotes mitochondrial biosynthesis, maintains mitochondrial function, and reduces cell apoptosis (179, 180). Meanwhile, HIF-1α is involved in the regulation of mitochondrial biogenesis and the modulation of nucleus-mitochondria communication independently of PGC-1α (181). Li et al. also introduced a novel mechanism in cell assays and an orthotopic transplantation model whereby SIRT1 enhances PGC-1α-mediated mitochondrial biogenesis and increases cell metabolism (182); While AMPK also enhances mitochondrial biogenesis and oxidative metabolism by promoting the transcriptional activity of PGC1α (183). Hypoxic microenvironment might inhibit the activation of AMPK targets, as well as apoptosis, by decreasing PGC-1α through SIRT1 deacetylation-dependent mechanisms; which may regulate the cytotoxic response to cisplatin and doxorubicin by licensing an apoptotic process controlled by mitochondria in NSCLC (66).

Emerging evidence has suggested that epigenetic modification may mediate primary resistance and contribute to acquired resistance during immunotherapy through the profound effect on many aspects of antitumor immunity (94, 95). Vorinostat (an HDAC inhibitor) has been shown to epigenetically restore BCL-2 protein family functions, which in turn restores the sensitivity of EGFR-mutated and gefitinib-resistant NSCLC to gefitinib. Clinical trials investigating the performance of epigenetic targeting agents, such as histone deacetylation, combined with adaptive T-cell transfer in patients with hypoxia-driven acquired resistance to prior immunotherapy are ongoing (96). The acetylation state of histones results from an imbalance between the activities of various histone acetyl transferase and the functioning of HDACs such as SIRT1. Additionally, a study of ongoing immunometabolism-targeted clinical trial also performed anti-PD-1 plus/minus PPAR-γ agonist (rosiglitazone) in many solid tumors, such as non-small-cell lung carcinoma(NCT04114136) (41).

In general, we speculate that SIRT1/PGC-1α/PPAR-γ signaling may represent a molecular metabolic mechanism underlying hypoxia-induced chemoresistance in the NSCLC microenvironment, and targeting hypoxia-related metabolic adaptation may be a potential therapeutic strategy for overcoming chemoresistance in NSCLC.

## Conclusion

In summary, the current review focused on SIRT1/PGC-1α/PPAR-γ as a possible mechanism underlying hypoxia-induced chemoresistance in NSCLC at the epigenetic and metabolic microenvironment level. This review offers a preclinical proof-of-concept for the targeting of the SIRT1/PPAR-γ/PGC-1α signaling pathway. The heterogeneity of metabolic adaptation under hypoxia-induced acquisition of chemoresistance has, until recently, remained unappreciated. Targeting signaling pathways of cancer metabolic dependency under hypoxia microenvironment could be explored as a new therapeutic combination strategy for overcoming chemoresistance in NSCLC.

## Author Contributions

QZ, RX, QD, XL, XY, HL, and HYL prepared and reviewed the manuscript. All authors contributed to the article and approved the submitted version.

## Funding

This study was supported in part by a grant from Clinical Research Plan of SHDC (SHDC2020CR3085B) and Minhang Health Commission of Development Plan 2020MW47. QD was supported by Key Innovative Team of Shanghai Top-Level University Capacity Building in Clinical Pharmacy and Regulatory Science at Shanghai Medical College, Fudan University (Shanghai Municipal Education Commission, HJW-R-2019-66-19), and Shanghai “Rising Stars of Medical Talent” Youth Development Program Youth Medical Talents Clinical Pharmacist Program [SHWRS(2020)_087].

## Conflict of Interest

The authors declare that the research was conducted in the absence of any commercial or financial relationships that could be construed as a potential conflict of interest.

## Abbreviations

NSCLC, Non-small cell lung cancer; SCLC, Small cell lung cancer; EGFR, Epidermal growth factor receptor; EGFR-TKIs, EGFR tyrosine kinase-inhibitors; VEGF, Vascular endothelial growth factor; HDAC, Histone deacetylase; SIRT1, Sirtuin 1; PGC-1α, Peroxisome proliferator—activated receptor-γ coactivator-1α;PPAR-γ, Peroxisome proliferator activated receptor-γ; NAD+, Nicotinamide adenine dinucleotide; PPRE, PPAR-responsive element; AMPK, Adenosine monophosphate activated protein kinase; HIF-1α, Hypoxia inducible factor-1alpha; OXPHOS, Oxidative phosphorylation; ROS, Reactive oxygen species; TME, Tumour microenvironment; EMT, Epithelial–mesenchymal transition.

## References

[1] Feng RM Zong YN Cao SM Xu RH. Current Cancer Situation in China: Good or Bad News From the 2018 Global Cancer Statistics? Cancer Commun (Lond) (2019) 39:22. 10.1186/s40880-019-0368-6 31030667 PMC6487510

[2] Chen W Zheng R Baade PD Zhang S Zeng H Bray F. Cancer Statistics in China, 2015. CA Cancer J Clin (2016) 66:115–32. 10.3322/caac.21338 26808342

[3] Ettinger DS Aisner DL Wood DE Akerley W Bauman J Chang JY. NCCN Guidelines Insights: Non-Small Cell Lung Cancer, Version 5.2018. J Natl Compr Canc Netw (2018) 16:807–21. 10.6004/jnccn.2018.0062 30006423

[4] Ettinger DS Wood DE Aggarwal C Aisner DL Akerley W Bauman JR. NCCN Guidelines Insights: Non-Small Cell Lung Cancer, Version 1.2020. J Natl Compr Canc Netw (2019) 17:1464–72. 10.6004/jnccn.2019.0059 31805526

[5] Normanno N De Luca A Bianco C Strizzi L Mancino M Maiello MR. Epidermal Growth Factor Receptor (EGFR) Signaling in Cancer. Gene (2006) 366:2–16. 10.1016/j.gene.2005.10.018 16377102

[6] Singh D Attri BK Gill RK Bariwal J. Review on EGFR Inhibitors: Critical Updates. Mini Rev Med Chem (2016) 16:1134–66. 10.2174/1389557516666160321114917 26996617

[7] Tomasello C Baldessari C Napolitano M Orsi G Grizzi G Bertolini F. Resistance to EGFR Inhibitors in non-Small Cell Lung Cancer: Clinical Management and Future Perspectives. Crit Rev Oncol Hematol (2018) 123:149–61. 10.1016/j.critrevonc.2018.01.013 29482776

[8] Liang J Lu T Chen Z Zhan C Wang Q. Mechanisms of Resistance to Pemetrexed in non-Small Cell Lung Cancer. Transl Lung Cancer Res (2019) 8:1107–18. 10.21037/tlcr.2019.10.14 PMC6976363 32010588

[9] Köberle B Tomicic MT Usanova S Kaina B. Cisplatin Resistance: Preclinical Findings and Clinical Implications. Biochim Biophys Acta (2010) 1806:172–82. 10.1016/j.bbcan.2010.07.004 20647037

[10] Okamoto K Saito Y Narumi K Furugen A Iseki K Kobayashi M. Different Mechanisms of Cisplatin Resistance Development in Human Lung Cancer Cells. Biochem Biophys Res Commun (2020) 530:745–50. 10.1016/j.bbrc.2020.07.040 32782152

[11] Apicella M Giannoni E Fiore S Ferrari KJ Fernández-Pérez D Isella C. Increased Lactate Secretion by Cancer Cells Sustains Non-Cell-Autonomous Adaptive Resistance to MET and EGFR Targeted Therapies. Cell Metab (2018) 28:848–65. 10.1016/j.cmet.2018.08.006 30174307

[12] Tumbrink HL Heimsoeth A Sos ML. The Next Tier of EGFR Resistance Mutations in Lung Cancer. Oncogene (2021) 40:1–11. 10.1038/s41388-020-01510-w 33060857

[13] Matsuo N Azuma K Sakai K Hattori S Kawahara A Ishii H. Association of EGFR Exon 19 Deletion and EGFR-TKI Treatment Duration With Frequency of T790M Mutation in EGFR-Mutant Lung Cancer Patients. Sci Rep (2016) 6:36458. 10.1038/srep36458 27811988 PMC5095551

[14] Singh PK Silakari O. Chemotherapeutics-Resistance “Arms” Race: An Update on Mechanisms Involved in Resistance Limiting EGFR Inhibitors in Lung Cancer. Life Sci (2017) 186:25–32. 10.1016/j.lfs.2017.08.001 28782530

[15] Santoni-Rugiu E Melchior LC Urbanska EM Jakobsen JN Stricker K Grauslund M. Intrinsic Resistance to EGFR-Tyrosine Kinase Inhibitors in EGFR-Mutant Non-Small Cell Lung Cancer: Differences and Similarities With Acquired Resistance. Cancers (Basel) (2019) 11:923. 10.3390/cancers11070923 PMC6678669 31266248

[16] Maity S Pai KSR Nayak Y. Advances in Targeting EGFR Allosteric Site as Anti-NSCLC Therapy to Overcome the Drug Resistance. Pharmacol Rep (2020) 72:799–813. 10.1007/s43440-020-00131-0 32666476 PMC7381467

[17] Tripathi SK Pandey K Rengasamy KRR Biswal BK. Recent Updates on the Resistance Mechanisms to Epidermal Growth Factor Receptor Tyrosine Kinase Inhibitors and Resistance Reversion Strategies in Lung Cancer. Med Res Rev (2020) 40:2132–76. 10.1002/med.21700 32596830

[18] Boyero L Sánchez-Gastaldo A Alonso M Noguera-Uclés JF Molina-Pinelo S Bernabé-Caro R. Primary and Acquired Resistance to Immunotherapy in Lung Cancer: Unveiling the Mechanisms Underlying of Immune Checkpoint Blockade Therapy. Cancers (Basel) (2020) 12:3729. 10.3390/cancers12123729 PMC7763130 33322522

[19] Wang F Wang S Zhou Q. The Resistance Mechanisms of Lung Cancer Immunotherapy. Front Oncol (2020) 10:568059. 10.3389/fonc.2020.568059 33194652 PMC7606919

[20] Faubert B Solmonson A DeBerardinis RJ. Metabolic Reprogramming and Cancer Progression. Science (2020) 368:eaaw5473. 10.1126/science.aaw5473 32273439 PMC7227780

[21] Keith B Simon MC. Hypoxia-Inducible Factors, Stem Cells, and Cancer. Cell (2007) 129:465–72. 10.1016/j.cell.2007.04.019 PMC3150586 17482542

[22] Cosse JP Michiels C. Tumour Hypoxia Affects the Responsiveness of Cancer Cells to Chemotherapy and Promotes Cancer Progression. Anticancer Agents Med Chem (2008) 8:790–7. 10.2174/187152008785914798 18855580

[23] Luo X Li A Chi X Lin Y Liu X Zhang L. Hypoxia-Activated Prodrug Enabling Synchronous Chemotherapy and HIF-1α Downregulation for Tumor Treatment. Bioconjug Chem (2021) 32:983–90. 10.1021/acs.bioconjchem.1c00131 33847488

[24] Hoogsteen IJ Marres HA van den Hoogen FJ Rijken PF Lok J Bussink J. Expression of EGFR Under Tumor Hypoxia: Identification of a Subpopulation of Tumor Cells Responsible for Aggressiveness and Treatment Resistance. Int J Radiat Oncol Biol Phys (2012) 84:807–14. 10.1016/j.ijrobp.2012.01.002 22420963

[25] Galluzzi L Senovilla L Vitale I Michels J Martins I Kepp O. Molecular Mechanisms of Cisplatin Resistance. Oncogene (2012) 31:1869–83. 10.1038/onc.2011.384 21892204

[26] Tong CWS Wu WKK Loong HHF Cho WCS To KKW. Drug Combination Approach to Overcome Resistance to EGFR Tyrosine Kinase Inhibitors in Lung Cancer. Cancer Lett (2017) 405:100–10. 10.1016/j.canlet.2017.07.023 28774798

[27] Scanlon SE Glazer PM. Multifaceted Control of DNA Repair Pathways by the Hypoxic Tumor Microenvironment. DNA Repair (Amst) (2015) 32:180–9. 10.1016/j.dnarep.2015.04.030 PMC4522377 25956861

[28] Masoud GN Li W. HIF-1α Pathway: Role, Regulation and Intervention for Cancer Therapy. Acta Pharm Sin B (2015) 5:378–89. 10.1016/j.apsb.2015.05.007 PMC4629436 26579469

[29] Gao XZ Wang GN Zhao WG Han J Diao CY Wang XH. Blocking OLFM4/HIF-1α Axis Alleviates Hypoxia-Induced Invasion, Epithelial-Mesenchymal Transition, and Chemotherapy Resistance in non-Small-Cell Lung Cancer. J Cell Physiol (2019) 1–9. 10.1002/jcp.28144 30680718

[30] Wang D Zhao C Xu F Zhang A Jin M Zhang K. Cisplatin-Resistant NSCLC Cells Induced by Hypoxia Transmit Resistance to Sensitive Cells Through Exosomal PKM2. Theranostics (2021) 11:2860–75. 10.7150/thno.51797 PMC7806469 33456577

[31] Yu HM Wang TC. Mechanism of Cisplatin Resistance in Human Urothelial Carcinoma Cells. Food Chem Toxicol (2012) 50:1226–37. 10.1016/j.fct.2012.01.040 22326969

[32] Wohlkoenig C Leithner K Deutsch A Hrzenjak A Olschewski A Olschewski H. Hypoxia-Induced Cisplatin Resistance Is Reversible and Growth Rate Independent in Lung Cancer Cells. Cancer Lett (2011) 308:134–43. 10.1016/j.canlet.2011.03.014 21669489

[33] Del Bello B Toscano M Moretti D Maellaro E. Cisplatin-Induced Apoptosis Inhibits Autophagy, Which Acts as a Pro-Survival Mechanism in Human Melanoma Cells. PloS One (2013) 8:e57236. 10.1371/journal.pone.0057236 23437349 PMC3577730

[34] Harhaji-Trajkovic L Vilimanovich U Kravic-Stevovic T Bumbasirevic V Trajkovic V. AMPK-Mediated Autophagy Inhibits Apoptosis in Cisplatin-Treated Tumour Cells. J Cell Mol Med (2009) 13:3644–54. 10.1111/j.1582-4934.2009.00663.x PMC4516513 20196784

[35] Wu HM Jiang ZF Ding PS Shao LJ Liu RY. Hypoxia-Induced Autophagy Mediates Cisplatin Resistance in Lung Cancer Cells. Sci Rep (2015) 5:12291. 10.1038/srep12291 26201611 PMC4511870

[36] Chen Y Henson ES Xiao W Huang D McMillan-Ward EM Israels SJ. Tyrosine Kinase Receptor EGFR Regulates the Switch in Cancer Cells Between Cell Survival and Cell Death Induced by Autophagy in Hypoxia. Autophagy (2016) 12:1029–46. 10.1080/15548627.2016.1164357 PMC4922445 27166522

[37] Arvold ND Heidari P Kunawudhi A Sequist LV Mahmood U. Tumor Hypoxia Response After Targeted Therapy in EGFR-Mutant Non-Small Cell Lung Cancer: Proof of Concept for FMISO-PET. Technol Cancer Res Treat (2016) 15:234–42. 10.1177/1533034615574386 PMC4565779 25759424

[38] Guo Q Lan F Yan X Xiao Z Wu Y Zhang Q. Hypoxia Exposure Induced Cisplatin Resistance Partially *via* Activating P53 and Hypoxia Inducible Factor-1α in Non-Small Cell Lung Cancer A549 Cells. Oncol Lett (2018) 16:801–8. 10.3892/ol.2018.8767 PMC6019907 29971135

[39] Pandey N Tyagi G Kaur P Pradhan S Rajam MV Srivastava T. Allicin Overcomes Hypoxia Mediated Cisplatin Resistance in Lung Cancer Cells Through ROS Mediated Cell Death Pathway and by Suppressing Hypoxia Inducible Factors. Cell Physiol Biochem (2020) 54:748–66. 10.33594/000000253 32809300

[40] Deben C Deschoolmeester V De Waele J Jacobs J Van den Bossche J Wouters A. Hypoxia-Induced Cisplatin Resistance in Non-Small Cell Lung Cancer Cells Is Mediated by HIF-1α and Mutant P53 and Can Be Overcome by Induction of Oxidative Stress. Cancers (Basel) (2018) 10:126. 10.3390/cancers10040126 PMC5923381 29690507

[41] Talty R Olino K. Metabolism of Innate Immune Cells in Cancer. Cancers (Basel) (2021) 13:904. 10.3390/cancers13040904 33670082 PMC7927092

[42] Noël G Langouo Fontsa M Willard-Gallo K. The Impact of Tumor Cell Metabolism on T Cell-Mediated Immune Responses and Immuno-Metabolic Biomarkers in Cancer. Semin Cancer Biol (2018) 52:66–74. 10.1016/j.semcancer.2018.03.003 29574171

[43] Lu Y Liu Y Oeck S Zhang GJ Schramm A Glazer PM. Hypoxia Induces Resistance to EGFR Inhibitors in Lung Cancer Cells *via* Upregulation of FGFR1 and the MAPK Pathway. Cancer Res (2020) 80:4655–67. 10.1158/0008-5472.Can-20-1192 PMC7642024 32873635

[44] Tian W Cao C Shu L Wu F. Anti-Angiogenic Therapy in the Treatment of Non-Small Cell Lung Cancer. Onco Targets Ther (2020) 13:12113–29. 10.2147/ott.S276150 PMC7699985 33262610

[45] Nilsson MB Robichaux J Herynk MH Cascone T Le X Elamin Y. Altered Regulation of HIF-1α in Naive- and Drug-Resistant EGFR-Mutant NSCLC: Implications for a Vascular Endothelial Growth Factor-Dependent Phenotype. J Thorac Oncol (2021) 16:439–51. 10.1016/j.jtho.2020.11.022 PMC8207565 33309987

[46] Horvath L Thienpont B Zhao L Wolf D Pircher A. Overcoming Immunotherapy Resistance in Non-Small Cell Lung Cancer (NSCLC) - Novel Approaches and Future Outlook. Mol Cancer (2020) 19:141. 10.1186/s12943-020-01260-z 32917214 PMC7488475

[47] Zhang WC Wells JM Chow KH Huang H Yuan M Saxena T. miR-147b-Mediated TCA Cycle Dysfunction and Pseudohypoxia Initiate Drug Tolerance to EGFR Inhibitors in Lung Adenocarcinoma. Nat Metab (2019) 1:460–74. 10.1038/s42255-019-0052-9 PMC6750230 31535082

[48] Calin GA Pardini B. Mir-Roring Hypoxia in EGFR-TKI Tolerance. Nat Metab (2019) 1:418–9. 10.1038/s42255-019-0057-4 32694876

[49] Lu Y Liu Y Oeck S Glazer PM. Hypoxia Promotes Resistance to EGFR Inhibition in NSCLC Cells *via* the Histone Demethylases, LSD1 and PLU-1. Mol Cancer Res (2018) 16:1458–69. 10.1158/1541-7786.Mcr-17-0637 PMC6170700 29934325

[50] Minakata K Takahashi F Nara T Hashimoto M Tajima K Murakami A. Hypoxia Induces Gefitinib Resistance in non-Small-Cell Lung Cancer With Both Mutant and Wild-Type Epidermal Growth Factor Receptors. Cancer Sci (2012) 103:1946–54. 10.1111/j.1349-7006.2012.02408.x PMC7659171 22863020

[51] Hapke RY Haake SM. Hypoxia-Induced Epithelial to Mesenchymal Transition in Cancer. Cancer Lett (2020) 487:10–20. 10.1016/j.canlet.2020.05.012 32470488 PMC7336507

[52] Shen X Zhi Q Wang Y Li Z Zhou J Huang J. Hypoxia Induces Multidrug Resistance *via* Enhancement of Epidermal Growth Factor-Like Domain 7 Expression in Non-Small Lung Cancer Cells. Chemotherapy (2017) 62:172–80. 10.1159/000456066 28351036

[53] Belisario DC Kopecka J Pasino M Akman M De Smaele E Donadelli M. Hypoxia Dictates Metabolic Rewiring of Tumors: Implications for Chemoresistance. Cells (2020) 9:2598. 10.3390/cells9122598 PMC7761956 33291643

[54] Li F Simon MC. Cancer Cells Don’t Live Alone: Metabolic Communication Within Tumor Microenvironments. Dev Cell (2020) 54:183–95. 10.1016/j.devcel.2020.06.018 PMC7375918 32640203

[55] Salem A Asselin MC Reymen B Jackson A Lambin P West CML. Targeting Hypoxia to Improve Non-Small Cell Lung Cancer Outcome. J Natl Cancer Inst (2018) 110:14–30. 10.1093/jnci/djx160 28922791

[56] Ziółkowska-Suchanek I. Mimicking Tumor Hypoxia in Non-Small Cell Lung Cancer Employing Three-Dimensional In Vitro Models. Cells (2021) 10:141. 10.3390/cells10010141 33445709 PMC7828188

[57] Hsu CC Shi J Yuan C Zhao D Jiang S Lyu J. Recognition of Histone Acetylation by the GAS41 YEATS Domain Promotes H2A.Z Deposition in non-Small Cell Lung Cancer. Genes Dev (2018) 32:58–69. 10.1101/gad.303784.117 29437725 PMC5828395

[58] Mi W Guan H Lyu J Zhao D Xi Y Jiang S. YEATS2 Links Histone Acetylation to Tumorigenesis of Non-Small Cell Lung Cancer. Nat Commun (2017) 8:1088. 10.1038/s41467-017-01173-4 29057918 PMC5651844

[59] Zhu S Dong Z Ke X Hou J Zhao E Zhang K. The Roles of Sirtuins Family in Cell Metabolism During Tumor Development. Semin Cancer Biol (2019) 57:59–71. 10.1016/j.semcancer.2018.11.003 30453040

[60] Chen X Sun K Jiao S Cai N Zhao X Zou H. High Levels of SIRT1 Expression Enhance Tumorigenesis and Associate With a Poor Prognosis of Colorectal Carcinoma Patients. Sci Rep (2014) 4:7481. 10.1038/srep07481 25500546 PMC4265776

[61] Yuan H Su L Chen WY. The Emerging and Diverse Roles of Sirtuins in Cancer: A Clinical Perspective. Onco Targets Ther (2013) 6:1399–416. 10.2147/ott.S37750 PMC3797239 24133372

[62] Yang H Bi Y Xue L Wang J Lu Y Zhang Z. Multifaceted Modulation of SIRT1 in Cancer and Inflammation. Crit Rev Oncog (2015) 20:49–64. 10.1615/critrevoncog.2014012374 25746104

[63] Shuang T Wang M Zhou Y Shi C. Over-Expression of Sirt1 Contributes to Chemoresistance and Indicates Poor Prognosis in Serous Epithelial Ovarian Cancer (EOC). Med Oncol (2015) 32:260. 10.1007/s12032-015-0706-8 26520143

[64] Chen H Zhang W Cheng X Guo L Xie S Ma Y. β2-AR Activation Induces Chemoresistance by Modulating P53 Acetylation Through Upregulating Sirt1 in Cervical Cancer Cells. Cancer Sci (2017) 108:1310–7. 10.1111/cas.13275 PMC5497720 28498637

[65] Yan L Zhao Q Liu L Jin N Wang S Zhan X. Expression of SIRT1 and Survivin Correlates With Poor Prognosis in Esophageal Squamous Cell Carcinoma. Med (Baltimore) (2020) 99:e21645. 10.1097/md.0000000000021645 PMC7447426 32846774

[66] Grbesa I Pajares MJ Martínez-Terroba E Agorreta J Mikecin AM Larráyoz M. Expression of Sirtuin 1 and 2 Is Associated With Poor Prognosis in Non-Small Cell Lung Cancer Patients. PloS One (2015) 10:e0124670. 10.1371/journal.pone.0124670 25915617 PMC4411155

[67] Chen X Hokka D Maniwa Y Ohbayashi C Itoh T Hayashi Y. Sirt1 Is a Tumor Promoter in Lung Adenocarcinoma. Oncol Lett (2014) 8:387–93. 10.3892/ol.2014.2057 PMC4063576 24959282

[68] Zhang T Rong N Chen J Zou C Jing H Zhu X. SIRT1 Expression Is Associated With the Chemotherapy Response and Prognosis of Patients With Advanced NSCLC. PloS One (2013) 8:e79162. 10.1371/journal.pone.0079162 24223900 PMC3818418

[69] Chen Y Wang T Wang W Hu J Li R He S. Prognostic and Clinicopathological Significance of SIRT1 Expression in NSCLC: A Meta-Analysis. Oncotarget (2017) 8:62537–44. 10.18632/oncotarget.19244 PMC5617527 28977967

[70] Jiang W Hou L Wei J Du Y Zhao Y Deng X. Hsa-miR-217 Inhibits the Proliferation, Migration, and Invasion in Non-Small Cell Lung Cancer Cells Via Targeting SIRT1 and P53/KAI1 Signaling. Balkan Med J (2020) 37:208–14. 10.4274/balkanmedj.galenos.2020.2019.9.91 PMC7285661 32267139

[71] Buler M Andersson U Hakkola J. Who Watches the Watchmen? Regulation of the Expression and Activity of Sirtuins. FASEB J (2016) 30:3942–60. 10.1096/fj.201600410RR 27591175

[72] Poulose N Raju R. Sirtuin Regulation in Aging and Injury. Biochim Biophys Acta (2015) 1852:2442–55. 10.1016/j.bbadis.2015.08.017 PMC4682052 26303641

[73] Liu L Wang P Liu X He D Liang C Yu Y. Exogenous NAD(+) Supplementation Protects H9c2 Cardiac Myoblasts Against Hypoxia/Reoxygenation Injury *via* Sirt1-P53 Pathway. Fundam Clin Pharmacol (2014) 28:180–9. 10.1111/fcp.12016 23384296

[74] You J Cheng J Yu B Duan C Peng J. Baicalin, a Chinese Herbal Medicine, Inhibits the Proliferation and Migration of Human Non-Small Cell Lung Carcinoma (NSCLC) Cells, A549 and H1299, by Activating the SIRT1/AMPK Signaling Pathway. Med Sci Monit (2018) 24:2126–33. 10.12659/msm.909627 PMC5909419 29632297

[75] Yang F. The Expression and Mechanism of Sirt1 and AMPK in Nonsmall Cell Lung Cancer. J Buon (2018) 23:106–10. 29552768

[76] van der Horst A Tertoolen LG de Vries-Smits LM Frye RA Medema RH Burgering BM. FOXO4 Is Acetylated Upon Peroxide Stress and Deacetylated by the Longevity Protein Hsir2(SIRT1). J Biol Chem (2004) 279:28873–9. 10.1074/jbc.M401138200 15126506

[77] Kops GJ Dansen TB Polderman PE Saarloos I Wirtz KW Coffer PJ. Forkhead Transcription Factor FOXO3a Protects Quiescent Cells From Oxidative Stress. Nature (2002) 419:316–21. 10.1038/nature01036 12239572

[78] Shin DH Choi YJ Park JW. SIRT1 and AMPK Mediate Hypoxia-Induced Resistance of Non-Small Cell Lung Cancers to Cisplatin and Doxorubicin. Cancer Res (2014) 74:298–308. 10.1158/0008-5472.Can-13-2620 24240701

[79] Mao B Hu F Cheng J Wang P Xu M Yuan F. SIRT1 Regulates YAP2-Mediated Cell Proliferation and Chemoresistance in Hepatocellular Carcinoma. Oncogene (2014) 33:1468–74. 10.1038/onc.2013.88 23542177

[80] Mvunta DH Miyamoto T Asaka R Yamada Y Ando H Higuchi S. SIRT1 Regulates the Chemoresistance and Invasiveness of Ovarian Carcinoma Cells. Transl Oncol (2017) 10:621–31. 10.1016/j.tranon.2017.05.005 PMC5491457 28667895

[81] Chen HC Jeng YM Yuan RH Hsu HC Chen YL. SIRT1 Promotes Tumorigenesis and Resistance to Chemotherapy in Hepatocellular Carcinoma and Its Expression Predicts Poor Prognosis. Ann Surg Oncol (2012) 19:2011–9. 10.1245/s10434-011-2159-4 22146883

[82] Yousafzai NA Zhou Q Xu W Shi Q Xu J Feng L. SIRT1 Deacetylated and Stabilized XRCC1 to Promote Chemoresistance in Lung Cancer. Cell Death Dis (2019) 10:363. 10.1038/s41419-019-1592-3 31043584 PMC6494911

[83] Han L Long Q Li S Xu Q Zhang B Dou X. Senescent Stromal Cells Promote Cancer Resistance Through SIRT1 Loss-Potentiated Overproduction of Small Extracellular Vesicles. Cancer Res (2020) 80:3383–98. 10.1158/0008-5472.Can-20-0506 PMC7611217 32366480

[84] Cao B Shi Q Wang W. Higher Expression of SIRT1 Induced Resistance of Esophageal Squamous Cell Carcinoma Cells to Cisplatin. J Thorac Dis (2015) 7:711–9. 10.3978/j.issn.2072-1439.2015.04.01 PMC4419326 25973238

[85] Lynch CJ Shah ZH Allison SJ Ahmed SU Ford J Warnock LJ. SIRT1 Undergoes Alternative Splicing in a Novel Auto-Regulatory Loop With P53. PloS One (2010) 5:e13502. 10.1371/journal.pone.0013502 20975832 PMC2958826

[86] Vaziri H Dessain SK Ng Eaton E Imai SI Frye RA Pandita TK. Hsir2(SIRT1) Functions as an NAD-Dependent P53 Deacetylase. Cell (2001) 107:149–59. 10.1016/s0092-8674(01)00527-x 11672523

[87] Eroglu Z Erdem C Oktem G Bozok Cetintas V Duzgun Z. Effect of SIRT1 Activators and Inhibitors on CD44+/CD133+−Enriched Non−Small Cell Lung Cancer Cells. Mol Med Rep (2020) 22:575–81. 10.3892/mmr.2020.11113 32377734

[88] Saeed MEM Rahama M Kuete V Dawood M Elbadawi M Sugimoto Y. Collateral Sensitivity of Drug-Resistant ABCB5- and Mutation-Activated EGFR Overexpressing Cells Towards Resveratrol Due to Modulation of SIRT1 Expression. Phytomedicine (2019) 59:152890. 10.1016/j.phymed.2019.152890 30921566

[89] Gong J Wang H Lou W Wang G Tao H Wen H. Associations of Sirtuins With Clinicopathological Parameters and Prognosis in Non-Small Cell Lung Cancer. Cancer Manag Res (2018) 10:3341–56. 10.2147/CMAR.S166946 PMC6138963 30237737

[90] Cao B He X Wang W Shi M. SIRT1 Influences the Sensitivity of A549 Non-Small Cell Lung Cancer Cell Line to Cisplatin *via* Modulating the Noxa Expression. Zhongguo Fei Ai Za Zhi (2016) 19:57–63. 10.3779/j.issn.1009-3419.2016.02.01 26903157 PMC6015143

[91] Zhang N Liang C Song W Tao D Yao J Wang S. Antitumor Activity of Histone Deacetylase Inhibitor Chidamide Alone or in Combination With Epidermal Growth Factor Receptor Tyrosine Kinase Inhibitor Icotinib in NSCLC. J Cancer (2019) 10:1275–87. 10.7150/jca.28570 PMC6400687 30854137

[92] Zang H Qian G Zong D Fan S Owonikoko TK Ramalingam SS. Overcoming Acquired Resistance of Epidermal Growth Factor Receptor-Mutant Non-Small Cell Lung Cancer Cells to Osimertinib by Combining Osimertinib With the Histone Deacetylase Inhibitor Panobinostat (LBH589). Cancer (2020) 126:2024–33. 10.1002/cncr.32744 PMC7241261 31999837

[93] Qin J Liu Y Lu Y Liu M Li M Li J. Hypoxia-Inducible Factor 1 Alpha Promotes Cancer Stem Cells-Like Properties in Human Ovarian Cancer Cells by Upregulating SIRT1 Expression. Sci Rep (2017) 7:10592. 10.1038/s41598-017-09244-8 28878214 PMC5587562

[94] Shien K Toyooka S Yamamoto H Soh J Jida M Thu KL. Acquired Resistance to EGFR Inhibitors Is Associated With a Manifestation of Stem Cell-Like Properties in Cancer Cells. Cancer Res (2013) 73:3051–61. 10.1158/0008-5472.Can-12-4136 PMC4506773 23542356

[95] Hashida S Yamamoto H Shien K Miyoshi Y Ohtsuka T Suzawa K. Acquisition of Cancer Stem Cell-Like Properties in Non-Small Cell Lung Cancer With Acquired Resistance to Afatinib. Cancer Sci (2015) 106:1377–84. 10.1111/cas.12749 PMC4638008 26202045

[96] Sun J Li G Liu Y Ma M Song K Li H. Targeting Histone Deacetylase SIRT1 Selectively Eradicates EGFR TKI-Resistant Cancer Stem Cells *via* Regulation of Mitochondrial Oxidative Phosphorylation in Lung Adenocarcinoma. Neoplasia (2020) 22:33–46. 10.1016/j.neo.2019.10.006 31765940 PMC6881627

[97] Edatt L Poyyakkara A Raji GR Ramachandran V Shankar SS Kumar VBS. Role of Sirtuins in Tumor Angiogenesis. Front Oncol (2019) 9:1516. 10.3389/fonc.2019.01516 32010617 PMC6978795

[98] Swinson DE O’Byrne KJ. Interactions Between Hypoxia and Epidermal Growth Factor Receptor in non-Small-Cell Lung Cancer. Clin Lung Cancer (2006) 7:250–6. 10.3816/CLC.2006.n.002 16512978

[99] Jensen KS Binderup T Jensen KT Therkelsen I Borup R Nilsson E. FoxO3A Promotes Metabolic Adaptation to Hypoxia by Antagonizing Myc Function. EMBO J (2011) 30:4554–70. 10.1038/emboj.2011.323 PMC3243591 21915097

[100] Singh V Ubaid S. Role of Silent Information Regulator 1 (SIRT1) in Regulating Oxidative Stress and Inflammation. Inflammation (2020) 43:1589–98. 10.1007/s10753-020-01242-9 32410071

[101] Wang Z Guo W Yi F Zhou T Li X Feng Y. The Regulatory Effect of SIRT1 on Extracellular Microenvironment Remodeling. Int J Biol Sci (2021) 17:89–96. 10.7150/ijbs.52619 33390835 PMC7757024

[102] Chen X Lu Y Zhang Z Wang J Yang H Liu G. Intercellular Interplay Between Sirt1 Signalling and Cell Metabolism in Immune Cell Biology. Immunology (2015) 145:455–67. 10.1111/imm.12473 PMC4515126 25890999

[103] Joo HY Yun M Jeong J Park ER Shin HJ Woo SR. SIRT1 Deacetylates and Stabilizes Hypoxia-Inducible Factor-1α (HIF-1α) *via* Direct Interactions During Hypoxia. Biochem Biophys Res Commun (2015) 462:294–300. 10.1016/j.bbrc.2015.04.119 25979359

[104] Chen R Dioum EM Hogg RT Gerard RD Garcia JA. Hypoxia Increases Sirtuin 1 Expression in a Hypoxia-Inducible Factor-Dependent Manner. J Biol Chem (2011) 286:13869–78. 10.1074/jbc.M110.175414 PMC3077588 21345792

[105] Yu Q Dong L Li Y Liu G. SIRT1 and HIF1α Signaling in Metabolism and Immune Responses. Cancer Lett (2018) 418:20–6. 10.1016/j.canlet.2017.12.035 29306019

[106] Yuan Y Cruzat VF Newsholme P Cheng J Chen Y Lu Y. Regulation of SIRT1 in Aging: Roles in Mitochondrial Function and Biogenesis. Mech Ageing Dev (2016) 155:10–21. 10.1016/j.mad.2016.02.003 26923269

[107] Lin QY Yin HL. RBM38 Induces SIRT1 Expression During Hypoxia in Non-Small Cell Lung Cancer Cells by Suppressing MIR34A Expression. Biotechnol Lett (2020) 42:35–44. 10.1007/s10529-019-02766-3 31760527

[108] Yu H Kim YM Cho M. Cytoplasm-Localized SIRT1 Downregulation Attenuates Apoptosis and Cell Cycle Arrest in Cisplatin-Resistant Lung Cancer A549 Cells. J Cancer (2020) 11:4495–509. 10.7150/jca.44383 PMC7255359 32489467

[109] Mamdani H Jalal SI. Histone Deacetylase Inhibition in Non-Small Cell Lung Cancer: Hype or Hope? Front Cell Dev Biol (2020) 8:582370. 10.3389/fcell.2020.582370 33163495 PMC7581936

[110] Lee P Chandel NS Simon MC. Cellular Adaptation to Hypoxia Through Hypoxia Inducible Factors and Beyond. Nat Rev Mol Cell Biol (2020) 21:268–83. 10.1038/s41580-020-0227-y PMC7222024 32144406

[111] Fahrmann JF Vykoukal JV Ostrin EJ. Amino Acid Oncometabolism and Immunomodulation of the Tumor Microenvironment in Lung Cancer. Front Oncol (2020) 10:276. 10.3389/fonc.2020.00276 32266129 PMC7105613

[112] Yoon JC Puigserver P Chen G Donovan J Wu Z Rhee J. Control of Hepatic Gluconeogenesis Through the Transcriptional Coactivator PGC-1. Nature (2001) 413:131–8. 10.1038/35093050 11557972

[113] Bargiela D Burr SP Chinnery PF. Mitochondria and Hypoxia: Metabolic Crosstalk in Cell-Fate Decisions. Trends Endocrinol Metab (2018) 29:249–59. 10.1016/j.tem.2018.02.002 29501229

[114] Zampieri LX Grasso D Bouzin C Brusa D Rossignol R Sonveaux P. Mitochondria Participate in Chemoresistance to Cisplatin in Human Ovarian Cancer Cells. Mol Cancer Res (2020) 18:1379–91. 10.1158/1541-7786.Mcr-19-1145 32471883

[115] Grasso D Zampieri LX Capelôa T Van de Velde JA Sonveaux P. Mitochondria in Cancer. Cell Stress (2020) 4:114–46. 10.15698/cst2020.06.221 PMC7278520 32548570

[116] Aventaggiato M Vernucci E Barreca F Russo MA Tafani M. Sirtuins’ Control of Autophagy and Mitophagy in Cancer. Pharmacol Ther (2021) 221:107748. 10.1016/j.pharmthera.2020.107748 33245993

[117] Ghosh P Vidal C Dey S Zhang L. Mitochondria Targeting as an Effective Strategy for Cancer Therapy. Int J Mol Sci (2020) 21:3363. 10.3390/ijms21093363 PMC7247703 32397535

[118] Sahinbegovic H Jelinek T Hrdinka M Bago JR Turi M Sevcikova T. Intercellular Mitochondrial Transfer in the Tumor Microenvironment. Cancers (Basel) (2020) 12:1787. 10.3390/cancers12071787 PMC7407231 32635428

[119] Dong LF Kovarova J Bajzikova M Bezawork-Geleta A Svec D Endaya B. Horizontal Transfer of Whole Mitochondria Restores Tumorigenic Potential in Mitochondrial DNA-Deficient Cancer Cells. Elife (2017) 6:e22187. 10.7554/eLife.22187 28195532 PMC5367896

[120] Pasquier J Guerrouahen BS Al Thawadi H Ghiabi P Maleki M Abu-Kaoud N. Preferential Transfer of Mitochondria From Endothelial to Cancer Cells Through Tunneling Nanotubes Modulates Chemoresistance. J Transl Med (2013) 11:94. 10.1186/1479-5876-11-94 23574623 PMC3668949

[121] Jones AW Yao Z Vicencio JM Karkucinska-Wieckowska A Szabadkai G. PGC-1 Family Coactivators and Cell Fate: Roles in Cancer, Neurodegeneration, Cardiovascular Disease and Retrograde Mitochondria-Nucleus Signalling. Mitochondrion (2012) 12:86–99. 10.1016/j.mito.2011.09.009 21983689

[122] Sen N Satija YK Das S. PGC-1α, a Key Modulator of P53, Promotes Cell Survival Upon Metabolic Stress. Mol Cell (2011) 44:621–34. 10.1016/j.molcel.2011.08.044 22099309

[123] Cruz-Bermúdez A Vicente-Blanco RJ Laza-Briviesca R García-Grande A Laine-Menéndez S Gutiérrez L. PGC-1alpha Levels Correlate With Survival in Patients With Stage III NSCLC and may Define a New Biomarker to Metabolism-Targeted Therapy. Sci Rep (2017) 7:16661. 10.1038/s41598-017-17009-6 29192176 PMC5709355

[124] Taguchi A Delgado O Celiktaş M Katayama H Wang H Gazdar AF. Proteomic Signatures Associated With P53 Mutational Status in Lung Adenocarcinoma. Proteomics (2014) 14:2750–9. 10.1002/pmic.201400378 PMC4403731 25331784

[125] Deng X Li Y Gu S Chen Y Yu B Su J. P53 Affects Pgc1α Stability Through AKT/GSK-3β to Enhance Cisplatin Sensitivity in Non-Small Cell Lung Cancer. Front Oncol (2020) 10:1252. 10.3389/fonc.2020.01252 32974127 PMC7471661

[126] Janani C Ranjitha Kumari BD. PPAR Gamma Gene–a Review. Diabetes Metab Syndr (2015) 9:46–50. 10.1016/j.dsx.2014.09.015 25450819

[127] Michalik L Desvergne B Wahli W. Peroxisome-Proliferator-Activated Receptors and Cancers: Complex Stories. Nat Rev Cancer (2004) 4:61–70. 10.1038/nrc1254 14708026

[128] Shi Y Hon M Evans RM. The Peroxisome Proliferator-Activated Receptor Delta, an Integrator of Transcriptional Repression and Nuclear Receptor Signaling. Proc Natl Acad Sci USA (2002) 99:2613–8. 10.1073/pnas.052707099 PMC122396 11867749

[129] Gou Q Gong X Jin J Shi J Hou Y. Peroxisome Proliferator-Activated Receptors (PPARs) Are Potential Drug Targets for Cancer Therapy. Oncotarget (2017) 8:60704–9. 10.18632/oncotarget.19610 PMC5601172 28948004

[130] Ban JO Kwak DH Oh JH Park EJ Cho MC Song HS. Suppression of NF-KappaB and GSK-3beta Is Involved in Colon Cancer Cell Growth Inhibition by the PPAR Agonist Troglitazone. Chem Biol Interact (2010) 188:75–85. 10.1016/j.cbi.2010.06.001 20540935

[131] Lee NJ Oh JH Ban JO Shim JH Lee HP Jung JK. 4-O-Methylhonokiol, a Pparγ Agonist, Inhibits Prostate Tumour Growth: P21-Mediated Suppression of NF-κb Activity. Br J Pharmacol (2013) 168:1133–45. 10.1111/j.1476-5381.2012.02235.x PMC3594673 23043610

[132] Remels AH Langen RC Gosker HR Russell AP Spaapen F Voncken JW. PPARgamma Inhibits NF-KappaB-Dependent Transcriptional Activation in Skeletal Muscle. Am J Physiol Endocrinol Metab (2009) 297:E174–83. 10.1152/ajpendo.90632.2008 19417127

[133] Han S Roman J. Peroxisome Proliferator-Activated Receptor Gamma: A Novel Target for Cancer Therapeutics? Anticancer Drugs (2007) 18:237–44. 10.1097/CAD.0b013e328011e67d 17264754

[134] Susaki Y Inoue M Minami M Sawabata N Shintani Y Nakagiri T. Inhibitory Effect of Pparγ on NR0B1 in Tumorigenesis of Lung Adenocarcinoma. Int J Oncol (2012) 41:1278–84. 10.3892/ijo.2012.1571 22843091

[135] Ni J Zhou LL Ding L Zhao X Cao H Fan F. Pparγ Agonist Efatutazone and Gefitinib Synergistically Inhibit the Proliferation of EGFR-TKI-Resistant Lung Adenocarcinoma Cells *via* the Pparγ/PTEN/Akt Pathway. Exp Cell Res (2017) 361:246–56. 10.1016/j.yexcr.2017.10.024 29080795

[136] Inoue K Kawahito Y Tsubouchi Y Yamada R Kohno M Hosokawa Y. Expression of Peroxisome Proliferator-Activated Receptor (PPAR)-Gamma in Human Lung Cancer. Anticancer Res (2001) 21:2471–6. 11724309

[137] Kim J Sato M Choi JW Kim HW Yeh BI Larsen JE. Nuclear Receptor Expression and Function in Human Lung Cancer Pathogenesis. PloS One (2015) 10:e0134842. 10.1371/journal.pone.0134842 26244663 PMC4526668

[138] Reka AK Goswami MT Krishnapuram R Standiford TJ Keshamouni VG. Molecular Cross-Regulation Between PPAR-γ and Other Signaling Pathways: Implications for Lung Cancer Therapy. Lung Cancer (2011) 72:154–9. 10.1016/j.lungcan.2011.01.019 PMC3075310 21354647

[139] Giaginis C Politi E Alexandrou P Sfiniadakis J Kouraklis G Theocharis S. Expression of Peroxisome Proliferator Activated Receptor-Gamma (PPAR-γ) in Human non-Small Cell Lung Carcinoma: Correlation With Clinicopathological Parameters, Proliferation and Apoptosis Related Molecules and Patients’ Survival. Pathol Oncol Res (2012) 18:875–83. 10.1007/s12253-012-9517-9 22426809

[140] Chang TH Szabo E. Induction of Differentiation and Apoptosis by Ligands of Peroxisome Proliferator-Activated Receptor Gamma in non-Small Cell Lung Cancer. Cancer Res (2000) 60:1129–38. 10706135

[141] Xu Y Jin J Zhang W Zhang Z Gao J Liu Q. EGFR/MDM2 Signaling Promotes NF-κb Activation *via* Pparγ Degradation. Carcinogenesis (2016) 37:215–22. 10.1093/carcin/bgv252 26718225

[142] Keshamouni VG Arenberg DA Reddy RC Newstead MJ Anthwal S Standiford TJ. PPAR-Gamma Activation Inhibits Angiogenesis by Blocking ELR+CXC Chemokine Production in Non-Small Cell Lung Cancer. Neoplasia (2005) 7:294–301. 10.1593/neo.04601 15799829 PMC1501135

[143] Reddy RC Srirangam A Reddy K Chen J Gangireddy S Kalemkerian GP. Chemotherapeutic Drugs Induce PPAR-Gamma Expression and Show Sequence-Specific Synergy With PPAR-Gamma Ligands in Inhibition of Non-Small Cell Lung Cancer. Neoplasia (2008) 10:597–603. 10.1593/neo.08134 18516296 PMC2386544

[144] Heudobler D Rechenmacher M Lüke F Vogelhuber M Pukrop T Herr W. Peroxisome Proliferator-Activated Receptors (PPAR)γ Agonists as Master Modulators of Tumor Tissue. Int J Mol Sci (2018) 19:3540. 10.3390/ijms19113540 PMC6274845 30424016

[145] Keshamouni VG Reddy RC Arenberg DA Joel B Thannickal VJ Kalemkerian GP. Peroxisome Proliferator-Activated Receptor-Gamma Activation Inhibits Tumor Progression in non-Small-Cell Lung Cancer. Oncogene (2004) 23:100–8. 10.1038/sj.onc.1206885 14712215

[146] Li M Lee TW Mok TS Warner TD Yim AP Chen GG. Activation of Peroxisome Proliferator-Activated Receptor-Gamma by Troglitazone (TGZ) Inhibits Human Lung Cell Growth. J Cell Biochem (2005) 96:760–74. 10.1002/jcb.20474 16149072

[147] Reddy AT Lakshmi SP Reddy RC. Pparγ as a Novel Therapeutic Target in Lung Cancer. PPAR Res (2016) 2016:8972570. 10.1155/2016/8972570 27698657 PMC5028876

[148] Yousefnia S Momenzadeh S Seyed Forootan F Ghaedi K Nasr Esfahani MH. The Influence of Peroxisome Proliferator-Activated Receptor γ (Pparγ) Ligands on Cancer Cell Tumorigenicity. Gene (2018) 649:14–22. 10.1016/j.gene.2018.01.018 29369787

[149] Shi S Yu G Huang B Mi Y Kang Y Simon JP. PPARG Could Work as a Valid Therapeutic Strategy for the Treatment of Lung Squamous Cell Carcinoma. PPAR Res (2020) 2020:2510951. 10.1155/2020/2510951 32565768 PMC7285416

[150] Zhao YZ Liu XL Shen GM Ma YN Zhang FL Chen MT. Hypoxia Induces Peroxisome Proliferator-Activated Receptor γ Expression *via* HIF-1-Dependent Mechanisms in HepG2 Cell Line. Arch Biochem Biophys (2014) 543:40–7. 10.1016/j.abb.2013.12.010 24374034

[151] Wang M Li G Yang Z Wang L Zhang L Wang T. Uncoupling Protein 2 Downregulation by Hypoxia Through Repression of Peroxisome Proliferator-Activated Receptor γ Promotes Chemoresistance of non-Small Cell Lung Cancer. Oncotarget (2017) 8:8083–94. 10.18632/oncotarget.14097 PMC5352384 28042952

[152] Cruz-Bermúdez A Laza-Briviesca R Vicente-Blanco RJ García-Grande A Coronado MJ Laine-Menéndez S. Cisplatin Resistance Involves a Metabolic Reprogramming Through ROS and PGC-1α in NSCLC Which Can Be Overcome by OXPHOS Inhibition. Free Radic Biol Med (2019) 135:167–81. 10.1016/j.freeradbiomed.2019.03.009 30880247

[153] Tan Z Luo X Xiao L Tang M Bode AM Dong Z. The Role of PGC1α in Cancer Metabolism and Its Therapeutic Implications. Mol Cancer Ther (2016) 15:774–82. 10.1158/1535-7163.MCT-15-0621 27197257

[154] Nishigaki A Kido T Kida N Kakita-Kobayashi M Tsubokura H Hisamatsu Y. Resveratrol Protects Mitochondrial Quantity by Activating SIRT1/PGC-1α Expression During Ovarian Hypoxia. Reprod Med Biol (2020) 19:189–97. 10.1002/rmb2.12323 PMC7138948 32273826

[155] Mastropasqua F Girolimetti G Shoshan M. Pgc1α: Friend or Foe in Cancer? Genes (Basel) (2018) 9:48. 10.3390/genes9010048 PMC5793199 29361779

[156] Chen W Wang Q Bai L Chen W Wang X Tellez CS. RIP1 Maintains DNA Integrity and Cell Proliferation by Regulating PGC-1α-Mediated Mitochondrial Oxidative Phosphorylation and Glycolysis. Cell Death Differ (2014) 21:1061–70. 10.1038/cdd.2014.25 PMC4207474 24583643

[157] Abdel-Wahab AF Mahmoud W Al-Harizy RM. Targeting Glucose Metabolism to Suppress Cancer Progression: Prospective of Anti-Glycolytic Cancer Therapy. Pharmacol Res (2019) 150:104511. 10.1016/j.phrs.2019.104511 31678210

[158] Huang CY Hsu LH Chen CY Chang GC Chang HW Hung YM. Inhibition of Alternative Cancer Cell Metabolism of EGFR Mutated Non-Small Cell Lung Cancer Serves as a Potential Therapeutic Strategy. Cancers (Basel) (2020) 12:181. 10.3390/cancers12010181 PMC7017237 31936895

[159] Picard F Kurtev M Chung N Topark-Ngarm A Senawong T Machado De Oliveira R. Sirt1 Promotes Fat Mobilization in White Adipocytes by Repressing PPAR-Gamma. Nature (2004) 429:771–6. 10.1038/nature02583 PMC2820247 15175761

[160] Meng X Tan J Li M Song S Miao Y Zhang Q. Sirt1: Role Under the Condition of Ischemia/Hypoxia. Cell Mol Neurobiol (2017) 37:17–28. 10.1007/s10571-016-0355-2 26971525 PMC11482061

[161] Yan X Qu X Tian R Xu L Jin X Yu S. Hypoxia-Induced NAD(+) Interventions Promote Tumor Survival and Metastasis by Regulating Mitochondrial Dynamics. Life Sci (2020) 259:118171. 10.1016/j.lfs.2020.118171 32738362

[162] Brooks CL Gu W. Ubiquitination, Phosphorylation and Acetylation: The Molecular Basis for P53 Regulation. Curr Opin Cell Biol (2003) 15:164–71. 10.1016/s0955-0674(03)00003-6 12648672

[163] Brunet A Sweeney LB Sturgill JF Chua KF Greer PL Lin Y. Stress-Dependent Regulation of FOXO Transcription Factors by the SIRT1 Deacetylase. Science (2004) 303:2011–5. 10.1126/science.1094637 14976264

[164] Han L Zhou R Niu J McNutt MA Wang P Tong T. SIRT1 Is Regulated by a PPAR{γ}-SIRT1 Negative Feedback Loop Associated With Senescence. Nucleic Acids Res (2010) 38:7458–71. 10.1093/nar/gkq609 PMC2995042 20660480

[165] Do MT Kim HG Choi JH Jeong HG. Metformin Induces microRNA-34a to Downregulate the Sirt1/Pgc-1α/Nrf2 Pathway, Leading to Increased Susceptibility of Wild-Type P53 Cancer Cells to Oxidative Stress and Therapeutic Agents. Free Radic Biol Med (2014) 74:21–34. 10.1016/j.freeradbiomed.2014.06.010 24970682

[166] Lee SY Hur GY Jung KH Jung HC Lee SY Kim JH. PPAR-Gamma Agonist Increase Gefitinib’s Antitumor Activity Through PTEN Expression. Lung Cancer (2006) 51:297–301. 10.1016/j.lungcan.2005.10.010 16386327

[167] Chang TH Szabo E. Enhanced Growth Inhibition by Combination Differentiation Therapy With Ligands of Peroxisome Proliferator-Activated Receptor-Gamma and Inhibitors of Histone Deacetylase in Adenocarcinoma of the Lung. Clin Cancer Res (2002) 8:1206–12. 11948134

[168] Roth KG Mambetsariev I Kulkarni P Salgia R. The Mitochondrion as an Emerging Therapeutic Target in Cancer. Trends Mol Med (2020) 26:119–34. 10.1016/j.molmed.2019.06.009 PMC6938552 31327706

[169] Shiratsuki S Hara T Munakata Y Shirasuna K Kuwayama T Iwata H. Low Oxygen Level Increases Proliferation and Metabolic Changes in Bovine Granulosa Cells. Mol Cell Endocrinol (2016) 437:75–85. 10.1016/j.mce.2016.08.010 27519633

[170] Tello D Balsa E Acosta-Iborra B Fuertes-Yebra E Elorza A Ordóñez Á. Induction of the Mitochondrial NDUFA4L2 Protein by HIF-1α Decreases Oxygen Consumption by Inhibiting Complex I Activity. Cell Metab (2011) 14:768–79. 10.1016/j.cmet.2011.10.008 22100406

[171] Tirpe AA Gulei D Ciortea SM Crivii C Berindan-Neagoe I. Hypoxia: Overview on Hypoxia-Mediated Mechanisms With a Focus on the Role of HIF Genes. Int J Mol Sci (2019) 20:6140. 10.3390/ijms20246140 PMC6941045 31817513

[172] Jin Q Huang F Xu X He H Zhang Y. High Expression of Hypoxia Inducible Factor 1α Related With Acquired Resistant to EGFR Tyrosine Kinase Inhibitors in NSCLC. Sci Rep (2021) 11:1199. 10.1038/s41598-020-79801-1 33441708 PMC7806909

[173] Ham PB 3rd Raju R. Mitochondrial Function in Hypoxic Ischemic Injury and Influence of Aging. Prog Neurobiol (2017) 157:92–116. 10.1016/j.pneurobio.2016.06.006 27321753 PMC5161736

[174] Aquilano K Vigilanza P Baldelli S Pagliei B Rotilio G Ciriolo MR. Peroxisome Proliferator-Activated Receptor Gamma Co-Activator 1alpha (PGC-1alpha) and Sirtuin 1 (SIRT1) Reside in Mitochondria: Possible Direct Function in Mitochondrial Biogenesis. J Biol Chem (2010) 285:21590–9. 10.1074/jbc.M109.070169 PMC2898414 20448046

[175] Nemoto S Fergusson MM Finkel T. SIRT1 Functionally Interacts With the Metabolic Regulator and Transcriptional Coactivator PGC-1{Alpha}. J Biol Chem (2005) 280:16456–60. 10.1074/jbc.M501485200 15716268

[176] Ren Z He H Zuo Z Xu Z Wei Z Deng J. The Role of Different SIRT1-Mediated Signaling Pathways in Toxic Injury. Cell Mol Biol Lett (2019) 24:36. 10.1186/s11658-019-0158-9 31164908 PMC6543624

[177] Vellinga TT Borovski T de Boer VC Fatrai S van Schelven S Trumpi K. Sirt1/Pgc1α-Dependent Increase in Oxidative Phosphorylation Supports Chemotherapy Resistance of Colon Cancer. Clin Cancer Res (2015) 21:2870–9. 10.1158/1078-0432.Ccr-14-2290 25779952

[178] St-Pierre J Drori S Uldry M Silvaggi JM Rhee J Jäger S. Suppression of Reactive Oxygen Species and Neurodegeneration by the PGC-1 Transcriptional Coactivators. Cell (2006) 127:397–408. 10.1016/j.cell.2006.09.024 17055439

[179] Yun SH Han SH Park JI. Peroxisome Proliferator-Activated Receptor γ and PGC-1α in Cancer: Dual Actions as Tumor Promoter and Suppressor. PPAR Res (2018) 2018:6727421. 10.1155/2018/6727421 29599799 PMC5828371

[180] Simmons GE Jr Pruitt WM Pruitt K. Diverse Roles of SIRT1 in Cancer Biology and Lipid Metabolism. Int J Mol Sci (2015) 16:950–65. 10.3390/ijms16010950 PMC4307284 25569080

[181] Gomes AP Price NL Ling AJ Moslehi JJ Montgomery MK Rajman L. Declining NAD(+) Induces a Pseudohypoxic State Disrupting Nuclear-Mitochondrial Communication During Aging. Cell (2013) 155:1624–38. 10.1016/j.cell.2013.11.037 PMC4076149 24360282

[182] Li Y Xu S Li J Zheng L Feng M Wang X. SIRT1 Facilitates Hepatocellular Carcinoma Metastasis by Promoting PGC-1α-Mediated Mitochondrial Biogenesis. Oncotarget (2016) 7:29255–74. 10.18632/oncotarget.8711 PMC5045394 27081083

[183] Herzig S Shaw RJ. AMPK: Guardian of Metabolism and Mitochondrial Homeostasis. Nat Rev Mol Cell Biol (2018) 19:121–35. 10.1038/nrm.2017.95 PMC5780224 28974774

